# Activation of lipophagy ameliorates cadmium-induced neural tube defects via reducing low density lipoprotein cholesterol levels in mouse placentas

**DOI:** 10.1007/s10565-024-09885-2

**Published:** 2024-05-21

**Authors:** Yu-Feng Zhang, Shuang Zhang, Qing Ling, Wei Chang, Lu-Lu Tan, Jin Zhang, Yong-Wei Xiong, Hua-Long Zhu, Po Bian, Hua Wang

**Affiliations:** 1 Department of Toxicology, School of Public Health, Anhui Medical University, Hefei, China; 2 Key Laboratory of Environmental Toxicology of Anhui Higher Education Institutes, Hefei, China; 3 Teaching and Research Section of Nuclear Medicine, School of Basic Medical Sciences, Anhui Medical University, Hefei, China; 4 Key Laboratory of Population Health Across Life Cycle (Anhui Medical University), Ministry of Education of the People’s Republic of China, Hefei, China

**Keywords:** Cadmium, Neural tube defects, Placenta, Lipophagy, LDL-C

## Abstract

Neural tube defects (NTDs) represent a prevalent and severe category of congenital anomalies in humans. Cadmium (Cd) is an environmental teratogen known to cause fetal NTDs. However, its underlying mechanisms remain elusive. This study aims to investigate the therapeutic potential of lipophagy in the treatment of NTDs, providing valuable insights for future strategies targeting lipophagy activation as a means to mitigate NTDs.We successfully modeled NTDs by Cd exposure during pregnancy. RNA sequencing was employed to investigate the transcriptomic alterations and functional enrichment of differentially expressed genes in NTD placental tissues. Subsequently, pharmacological/genetic (*Atg5*^*-/-*^ placentas) experiments confirmed that inducing placental lipophagy can alleviate Cd induced-NTDs. We found that Cd exposure caused NTDs. Further analyzed transcriptomic data from the placentas with NTDs which revealed significant downregulation of low-density lipoprotein receptor associated protein 1(*Lrp1*) gene expression responsible for positive regulation of low-density lipoprotein cholesterol (LDL-C) transport. Correspondingly, there was an increase in maternal serum/placenta/amniotic fluid LDL-C content. Subsequently, we have discovered that Cd exposure activated placental lipophagy. Pharmacological/genetic (*Atg5-/-* placentas) experiments confirmed that inducing placental lipophagy can alleviate Cd induced-NTDs. Furthermore, our findings demonstrate that activation of placental lipophagy effectively counteracts the Cd-induced elevation in LDL-C levels. Lipophagy serves to mitigate Cd-induced NTDs by reducing LDL-C levels within mouse placentas.

**Supplementary Information:**

The online version contains supplementary material available at 10.1007/s10565-024-09885-2.

## Introduction

A neural tube defect (NTD) occurs when the neural folds fail to close during embryonic development, resulting in birth defects like anencephaly, spina bifida, and cerebral palsy (Fimia et al. 2007; Rhinn et al. 2022). The majority of individuals with NTDs die during or shortly after birth, or they suffer from varying degrees of disabilities (Rauch et al. 2020). The risk of developing NTDs significantly increases in individuals with a folic acid deficiency, as demonstrated by numerous studies (Santander et al. 2018; Tian et al. 2021). The use of folic acid as a new treatment for NTDs has emerged in recent years, but it cannot prevent all types of NTDs (Cao et al. 2022). Thus, there is an urgent imperative to establish a paradigm of NTDs in order to delve profoundly into the intricacies of its pathogenesis and unearth potential therapeutic targets, thereby facilitating the development of alternative preventive strategies.

Comprising a series of complex polygenic diseases, NTDs are caused synergistically by genetics and environment (Nandadasa et al. 2019; Yan et al. 2021). Environmental Cd exposure is positively associated with NTDs according to several epidemiological studies (Zhang et al. 2016; Zhang et al. 2004). Maternal Cd exposure results in NTDs in CD-1 mice (Yan et al. 2021). Importantly, we have shown that Cd accumulates at high levels in the placenta and impairs its structure and function, but rarely crosses the placental barrier into the fetus (Wang et al. 2016; Xiong et al. 2020). Researchers have found that placental defects cause fetal malformations and fetal death (Zhang et al. 2016).Thus, we hypothesized that Cd-evoked NTDs may be influenced by placental impairment. The placental etiological mechanism of NTDs caused by Cd exposure, however, remains elusive.

Disturbances of fetal neural tube development were significantly associated with human dyslipidemia, especially LDL-C levels in pregnancy (Buffart et al. 2008). Consequently, LDL cholesterol is commonly referred to as "bad" cholesterol. Recent studies have demonstrated a significant impact of maternal cholesterol transfer across the placenta on fetal cholesterol levels (Solis-Paredes et al. 2016). After exposure to Cd, C57/BL mice exhibited an elevation in serum LDL-C levels, thereby inducing parkinsonian syndrome through disruption of brain lipid metabolism (Xu et al. 2023). In TM3 cells, Cd treatment reduces the expression of low-density lipoprotein receptors, preventing the extracellular LDL-C from being broken down into free cholesterol (Zhou et al. 2022). Therefore, we hypothesized that Cd exposure during pregnancy induced NTDs by raising LDL cholesterol levels in mice. However, it is not clear how Cd exposure raises LDL-C levels.

Lipophagy, is a relatively recent discovery that selectively degrades lipid droplets (LDs), linked autophagy and lipid metabolism, it plays a vital role in cholesterol metabolism (Maan et al. 2018). Currently, only ATGL, PNPLA8 and OPR8 have been identified as lipophagy receptors in mammals (Li et al. 2021; Wang and Zhang 2023). LDs accumulate when lipophagy is inhibited, while LDs are degraded when lipophagy is activated (Maan et al. 2018; Zhang et al. 2023). The latest research findings, maternal Cd exposure during pregnancy activated placental trophoblast cells and mouse placental lipophagy (Zhang et al. 2023). Lipophagy has been found to reverse Cd-induced lipid accumulation in placental and hepatocytes, according to previous studies (Ye et al. 2020). Another study found that lipophagy activates the production of free cholesterol from degraded LDs to promote the synthesis of placental progesterone (Zhang et al. 2023). Therefore, we hypothesize that exposure to Cd has the potential to induce placental lipiophagy.

This study aims to investigate the potential placental etiological mechanism of Cd-induced NTDs. To gain deeper insights into this process, we conducted further verification through pharmacological, genetic, and transcriptomic approaches. We will gain valuable insight into Cd-induced NTDs and our understanding of their underlying mechanisms through these findings.

## Materials and methods

### Reagents

Protease Inhibitor Cocktail (HY-K0010, Med Chem Express), Rapamycin (an activator of lipophagy, Rap, 10219, Med Chem Express) and 3-methyladenine (an inhibitor of lipophagy, 3-MA, 19312, Med Chem Express). BCA Analysis Kit (23225, Thermo Fisher Scientific) and Chemiluminescence reagents (Cat, No.:34094, Thermo Fisher Scientific). Antibodies LC3B (3868s, CST), Actin (4970s, CST), Atg5 (ab108327, Abcam), LC3B (ab48394, Abcam), ATGL (55190-1-AP, Wuhan Sanying) and Lrp1(13213-1-AP, Wuhan Sanying). Cadmium chloride (CdCl2, 202908, Sigma-Aldrich).

### Animals

We purchased C57BL/6J mice (8-week-old, wild-type) from Vital River (Beijing, China). Two weeks of adaptive feeding in SPF conditions were given to all mice. Please refer to the animal section of the supplement materials for detailed feeding conditions. After two weeks of adaptive feeding, healthy two males and four females were mated in a cage at 8:30 p.m. The female mice underwent examination for vaginal plug at 7:00 a.m. The female mice underwent examination for vaginal emboli at 7:00 a.m. The presence of white vaginal emboli in female mice during morning checks indicated day 0 of gestation (GD0).

Experimental design 1: To establish a model of NTDs caused by Cd exposure during pregnancy. The pregnant mice were divided into control group, Low Cadmium group and High Cadmium group, with 12 mice in each group. On GD8, the mice in the Ctrl group (normal saline, i.p.), the LCd group (CdCl_2_,1.0 mg/kg, i.p.) and the HCd group (CdCl_2_, 2.0 mg/kg, i.p.). Please refer to the animal section of the supplement materials for selection of Cd dose. Experimental design 2: to investigate the effect of lipophagy on Cd-evoked NTDs, pregnant mice were treated with 3-MA (20 mg/kg/day, i.p.) (Liu et al. 2019) or Rap (0.1 mg/kg/day, i.p.) (Dai et al. 2021) from GD7 to GD15 after CdCl_2_ (2.0 mg/kg) exposure. Experimental design 3: *Dpp3-Cre/Atg5*^*flox/-*^ pregnant mice were administered CdCl_2_ (2.0 mg/kg) on GD8 (Zhang et al. 2023).

Placentas were collected from each corresponding fetus with NTDs and kept separately during the experiment. All the analyzed placentas were derived from fetuses with NTDs and that these fetuses came from different litters. All mice were anesthetized by tribromoethanol (100 mg/kg, MKCM1080, Sigma, i.p.) and then euthanized humanely. Please refer to the animal section of the supplement materials for detailed process of animal experiments.

### RNA sequencing and data analysis

The placental etiological mechanism of NTDs induced by Cd exposure was investigated by transcriptome sequencing. Placentas were collected according to whether the fetus had NTDs, and each placenta was placed in an enzyme-free EP tube. Three placentas from different litters were used in each group for RNA sequencing. No-NTDs placentas from different litters were included in the control group, each group included 3 placentas. NTDs placentas from different pregnant litters were included in high-dose cadmium (Cd, 2.0 mg/kg) group, each group included 3 placentas. Send it to the testing company for testing (Shanghai Kangcheng Biological Co., LTD). Please refer to the RNA sequencing and data analysis section of the supplement materials for detailed process of RNA sequencing.

### Immunoblotting

All the analyzed placentas were derived from fetuses with NTDs and that these fetuses came from different litters. Total protein was extracted using RIPA buffer (Protease Inhibitor Cocktail, HY-K0010, MedChemExpress). To measure protein concentration, the Pierce BCA Protein Analysis Kit was used as directed by the manufacturer. The placental lysates were electrophoretically separated using 12.5% SDS-PAGE and transferred to a PVDF membrane. Then, they were incubated with freshly prepared 5% skim milk at room temperature for 1.5 hours. Following that, PVDF membranes were incubated for an hour to three hours with primary antibodies. A secondary antibody was incubated for 1-2 hours on PVDF membranes after washing. Lastly, gray-scale imaging of PVDF membrane was performed using Chemiluminescence reagents (34094, Thermo Fisher Scientific) with a universal imaging system. The gray levels of the corresponding bands were analyzed and the amount of the target protein was calculated using Image J software. Please refer to the Immunoblotting and data analysis section of the Supplement materials for detailed immunoblotting procedures.

### Real-time qPCR

TRIzol (Ambion, 15596026) was used to extract mouse placental RNA. Reverse transcription of total RNA into cDNA was performed using an RT reagent kit (Roche, 04897030001). The qPCR system utilized in this study was the Roche Lightcyler 480 QPCR System (version 1.5.0) along with the SYBR Green PCR Master Mix (Roche, 06924204001). *18S* was used as the housekeeping gene. Primers sequences are given in table 1. The data analysis was conducted employing the 2^−ΔΔCt^ methodology.

**Table 1:** Primers for real-time RT-PCR

*ATGL*	Sequences (5’-3’)	Species
Forward	CTTGTGTCCTCCGCTTATGTC	Mouse
Reverse	GCAGAGGTCACGGTCTTCAC	*Mouse*
*ORP8*	Sequences (5’-3’)	Species
Forward	CCACGAAGGAGTTACTCAGCA	*Mouse*
Reverse	ATCAGAAGCACGCCAGGTTTC	*Mouse*
*PNPLA8*	Sequences (5’-3’)	Species
Forward	TCAAAGGCTATTTTTGGCAGTCA	*Mouse*
Reverse	CTCGACGGCTTGCTTAACATT	*Mouse*
*Lrp1*	Sequences (5’-3’)	Species
Forward	CCACTATGGATGCCCCTAAAAC	*Mouse*
Reverse	GCAATCTCTTTCACCGTCACA	*Mouse*
*Lrp2*	Sequences (5’-3’)	*Species*
Forward	GGCAGTGGGAATTTTCGCTG	Mouse
Reverse	CAGGAGCTAGGGATGCAGG	*Mouse*
*Lrp3*	Sequences (5’-3’)	Species
Forward	AGCACACTGAACGTCGAGG	*Mouse*
Reverse	TCACCACGGTCTCCTTGAATG	*Mouse*
*Lrp4*	Sequences (5’-3’)	Species
Forward	GCACACGGAATAGCCAGCA	*Mouse*
Reverse	GGATACAGGTACATTCGCCAAG	*Mouse*
*Lrp5*	Sequences (5’-3’)	Species
Forward	ACGTCCCGTAAGGTTCTCTTC	*Mouse*
Reverse	GCCAGTAAATGTCGGAGTCTAC	*Mouse*
*Lrp6*	Sequences (5’-3’)	Species
Forward	TGCAAACAGACGGGACTTGAG	*Mouse*
Reverse	CGGGGACAATAATCCAGAAACAA	*Mouse*
Lrp7	Sequences (5’-3’)	Species
Forward	CTTCCTGCCCATGACTGAGG	*Mouse*
Reverse	GACCCCAGACGCACAAAGTAG	*Mouse*
Lrp8	Sequences (5’-3’)	Species
Forward	GAGGACCAGTTTCGGTGTCG	*Mouse*
Reverse	GTTGTCCGAGCAGTCGTTGT	*Mouse*

### Measurement of low-density lipoprotein cholesterol (LDL-C) content

The content of LDL-C in maternal blood, placenta and amniotic fluid was measured. LDL-C in maternal serum and fetal amniotic fluid was measured by biochemical analyzer. The LDL-C test kit was procured from Zhejiang Yili Biotechnology Co., LTD., China. Subsequently, the fresh serum samples and amniotic fluid of mice were detected by biochemical analyzer. The placenta sample is measured as follows, in accordance with tissue mass (g): for ice bath homogenization, it is recommended to weigh approximately 0.1 g of tissue and add 1 mL of extraction liquid at a ratio of 1:5~10. After centrifugation at 4 °C for 10 min, the supernatant should be placed overnight on ice for further analysis. Each set contains six placenta samples, each placenta came from a different litter.

### TEM (Transmission Electron Microscopy)

1% glutaraldehyde was used to fix 1 mm^3^ of placental tissue. Samples were prepared at Electron Microscope Center of Anhui Medical University (Leica EM UC7, Germany). Observation of LD-containing autophagosomes: lipid droplets are encapsulated in autophagosomes in double or multilayer membranes. In LD-containing autophagosomes, lipid droplets are broken down.

### Immunofluorescence

The placentas from different litters in each group were collected. Followed by fixation with 4% paraformaldehyde and dehydration using a 30% sucrose solution. Subsequently, 5 μm placental sections were prepared from frozen placental tissue using a freezing microtome (Leica CM 1950). Frozen Section is a method of rapidly cooling tissue to a certain hardness under low temperature conditions, and then sectioning. Temperature is an important link in the preparation of frozen sections for sample fixation. Before freezing, adjust the temperature of the flash freezing head and the temperature in the box of the constant temperature freezing microtome to the appropriate slicing temperature, generally -25 °C to -18 °C. LC3B and ATGL antibodies were incubated with placental sections for 2 h at 37 °C. Fluorescein-conjugated secondary antibodies were incubated for 1.5 h at 37 °C after PBS washing. DAPI (BL739A, biosharp) was used for 8 minutes to label the nuclei. Each section was viewed using a laser confocal microscope.

### Lipid droplets (LDs) staining experiment

The Bodipy staining of lipid droplets uses placenta, which is different from the placenta used in molecular experiments. Subsequently, 5 μm placental sections were prepared from frozen placental tissue using a freezing microtome. PBS was used 3 times for 5 minutes each time to clean the sections. The placental tissue was then permeated for 1 h. After 15 minutes, the sections were dyed with freshly prepared Bodipy 493/503 (MED25592) solution. DAPI was used for 8 minutes to label the nuclei. Finally, the examination of each section was conducted utilizing a laser confocal microscope. The LDs areas in placental labyrinth were assessed using ImageJ (*n*=3).

### Statistical analysis

The data were expressed as *mean ± SD*. Following data analysis using GraphPad Prism9.0 software, we employed the *student's t-test* to compare the differences between the two groups. For multiple group comparisons, we used *one-way ANOVA* and assessed the normality and homogeneity of variance of each group's data before comparison. To further reduce errors, we used the *Bonferroni* method to correct the two-by-two comparison results. The data were entered into the GraphPad Prism9.0 software for analysis. The significance level was set at *P* < 0.05.

## Results

### Gestational Cd exposure induces NTDs

**Fig. 1:**
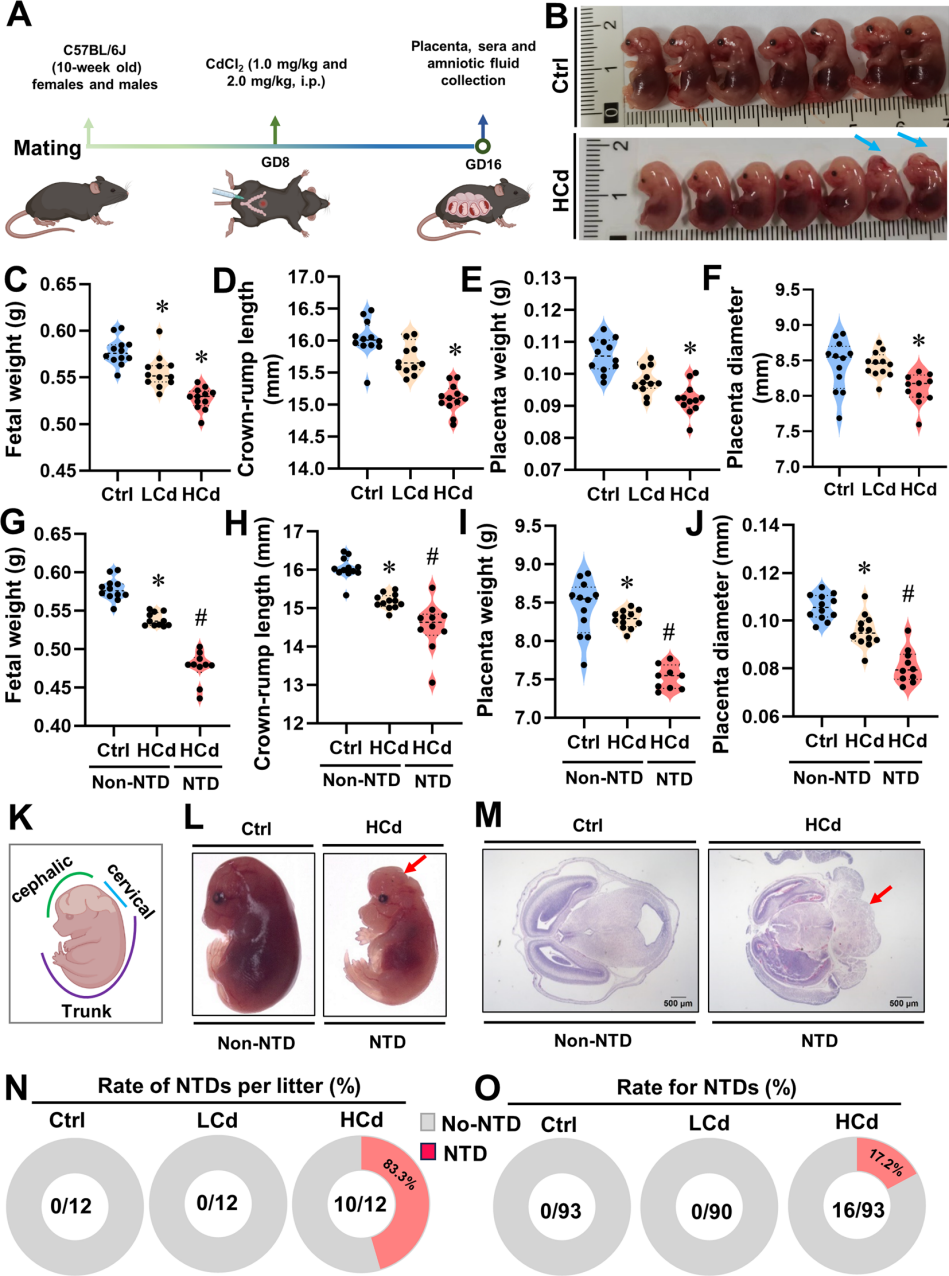
Gestational Cd exposure induces NTDs. Pregnant mice were intraperitoneally injected with normal saline (Ctrl), low-dose cadmium (LCd, 1.0 mg/kg), or high-dose cadmium (HCd, 2.0 mg/kg) on GD8, and fetal external malformations were observed on GD16, each group consisted of 12 litters. (**A**) Research design process. (**B**) Representative image of fetus mice in Ctrl group and HCd group. Blue arrows represent fetus with NTDs. (**C**) Fetal weight. (**D**) Crown-rump length. (**E**) Placenta weight. (**F**) Placenta diameter. (**G**) Fetal weight. (**H**) Crown-rump length. (**I**) Placenta weight. (**J**) Placenta diameter. (**K**) Fetal are divided into three parts: cephalic, cervical and trunk/tail. (**L**) The external malformations of fetal were observed by dissecting microscope. Red arrows represent failed to close during neurulation. (**M**) Embryonic neural tube H&E staining results in normal and NTDs. Scale bar, 500 μm. Red arrows represent fetus with NTDs. (**N**) Litters with NTDs. Divide litters with NTDs by total litters per group. (**O**) Rate for NTDs. Data were shown as *mean ± SD* (*n*=12). * *P* < 0.05, **P < 0.01 *vs*. Ctrl mice. ^#^ *P* < 0.05, ^##^ *P* < 0.01 *vs*. Cd (Non-NTD) mice

To investigate the impact of maternal exposure to Cd on the occurrence of NTDs, pregnant mice were subjected to Cd exposure on GD8 (Fig. 1A and B). Under stereomicroscopic observation, the fetus showed significant growth retardation and deformities after maternal exposure to HCd, and the brain vesicles showed dysplasia (Fig. 1K and L). The placentas of pregnant mice treated with CdCl_2_ (2.00 mg/kg) exhibited detectable levels of Cd at 0.432±0.02 μg/g (Fig. S1A). Maternal exposure to HCd significantly decreased fetal weight and crown-rump length, as well as placenta weight and diameter, particularly in embryos with NTDs compared to normal embryos (Fig. 1C-J). Hematoxylin-eosin staining revealed that the NTD embryos failed to close during neurulation compared to normal embryos (Fig. 1M). Exposure of pregnant mice to HCd resulted in an NTD rate of 17.3% (16/93) and 83.3% (10/12) of litters had NTDs, as depicted in Fig. 1N and O. The above results indicate that maternal HCd exposure leads to NTDs.

### Gestational Cd exposure downregulates expression of Lrp1 and increases the level of LDL-C in mouse placentas

**Fig. 2:**
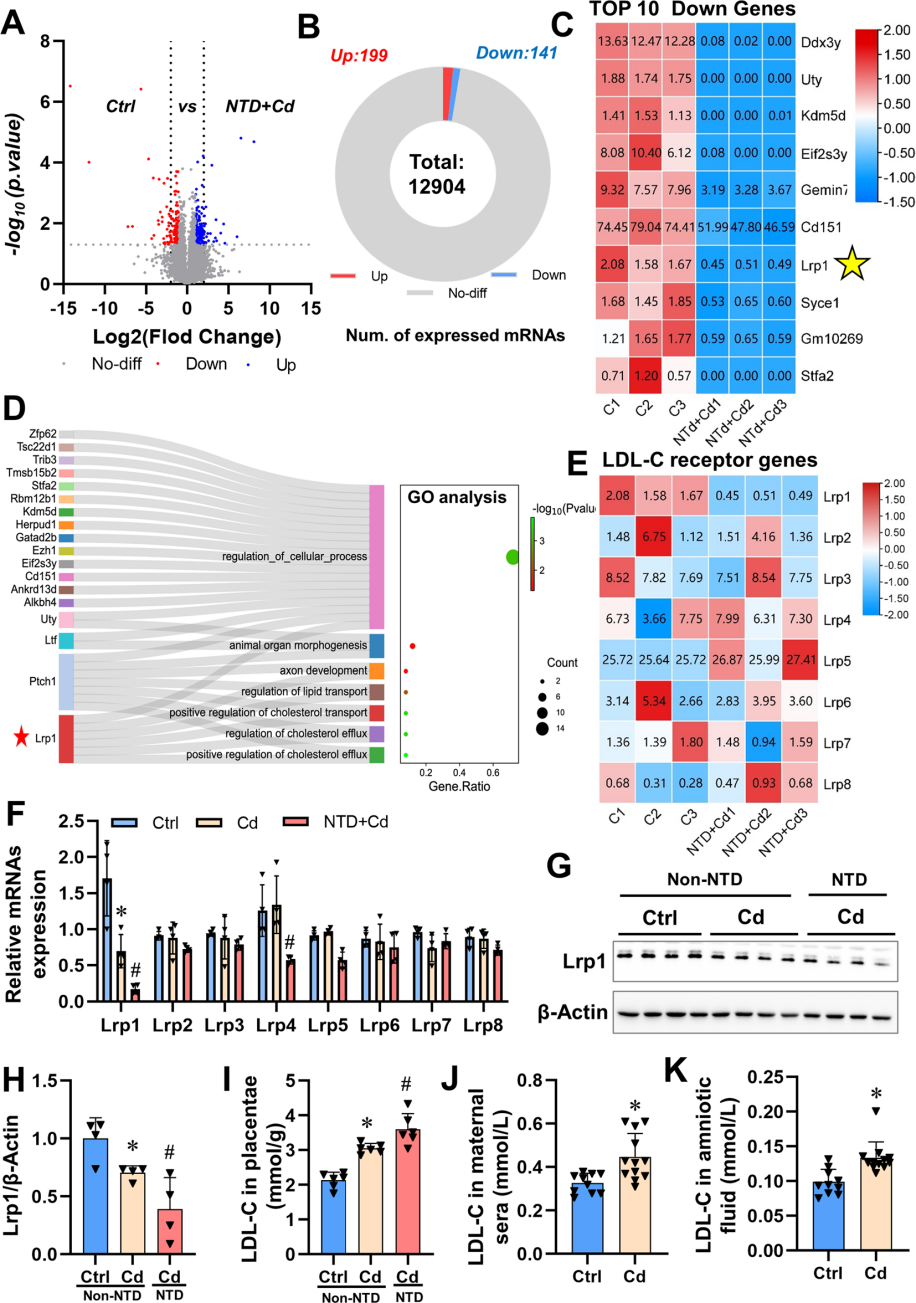
Gestational Cd exposure downregulates expression of Lrp1 and increases the level of LDL-C in mouse placentas. Pregnant mice were administered CdCl_2_ (2.0 mg/kg) on GD8 to establish a NTDs model. (**A**) Volcano plots, used to identify differentially expressed genes between Ctrl and NTD+Cd group placentas. (**B**) Volcano plot results statistics; (**C**) The expression trend of top 10 down genes in mouse placentas, the FPKM of gene in samples (*n*=3). (**D**) Enriched GO term pathways of top 20 down genes. (**E**) RNA sequencing of placenta and heatmap of low-density lipoprotein receptor-related protein family, the FPKM of gene in samples (*n*=3). (**F**) Relative mRNA levels of low-density lipoprotein receptor-related protein family genes in mouse placentae (*n*=4). (**G**) Lrp1 immunoblots from mouse placentae (*n*=4). (**H**) Quantification for Lrp1. (**I**) LDL-C in placentae (*n*=6). (**J**) LDL-C in maternal sera, in the Cd-treated group a litter of fetuses had some NTDS and some did not (*n*=12). (**K**) LDL-C in amniotic fluid, amniotic fluid was collected from a litter of fetuses (*n*=12). Data were shown as *mean ± SD*. * *P* < 0.05, **P < 0.01 *vs*. Ctrl mice. ^#^ *P* < 0.05, ^##^ *P* < 0.01 *vs*. Cd (Non-NTD) mice

To further investigate the mechanism by which maternal Cd exposure induces NTDs, we conducted transcriptomics analysis in mouse placentas with and without NTDs. After conducting a comprehensive analysis, we identified a total of 340 genes (199 up-regulated and 141 down-regulated) that exhibited significant differential expression compared to the Ctrl vs NTD+Cd group (Figs. 2A and B). The top 10 downregulated genes analysis showed that Cd exposure significantly decreased the expression of *Lrp1* in mouse placentae with NTDs (Figs. 2C). GO analysis showed that *Lrp1* gene was mainly enriched and presented in positive regulation of LDL-C transport and axon development (Fig. 2D). For mouse placentas, the expression of low-density lipoprotein receptor-related protein family was presented in the heatmap showed that Cd exposure significantly decreased the expression of *Lrp1* (Fig. 2E). Further analysis demonstrated that Cd exposure significantly reduced both mRNA and protein levels of Lrp1 in mouse placentas with NTDs (Figs. 2F-H), while increasing LDL-C levels in maternal sera, amniotic fluid, and placenta tissues from mice with NTDs induced by gestational Cd exposure (Figs. 2I-K). These results suggest that Cd exposure during pregnancy increases placental LDL-C levels by decreasing Lrp1 expression. Therefore, we selected LDL-C as a risk factor for NTDs for further analysis. These findings suggest that embryo NTDs caused by Cd exposure may be due to elevated levels of LDL cholesterol.

### Gestational Cd exposure activates placental lipophagy

**Fig 3:**
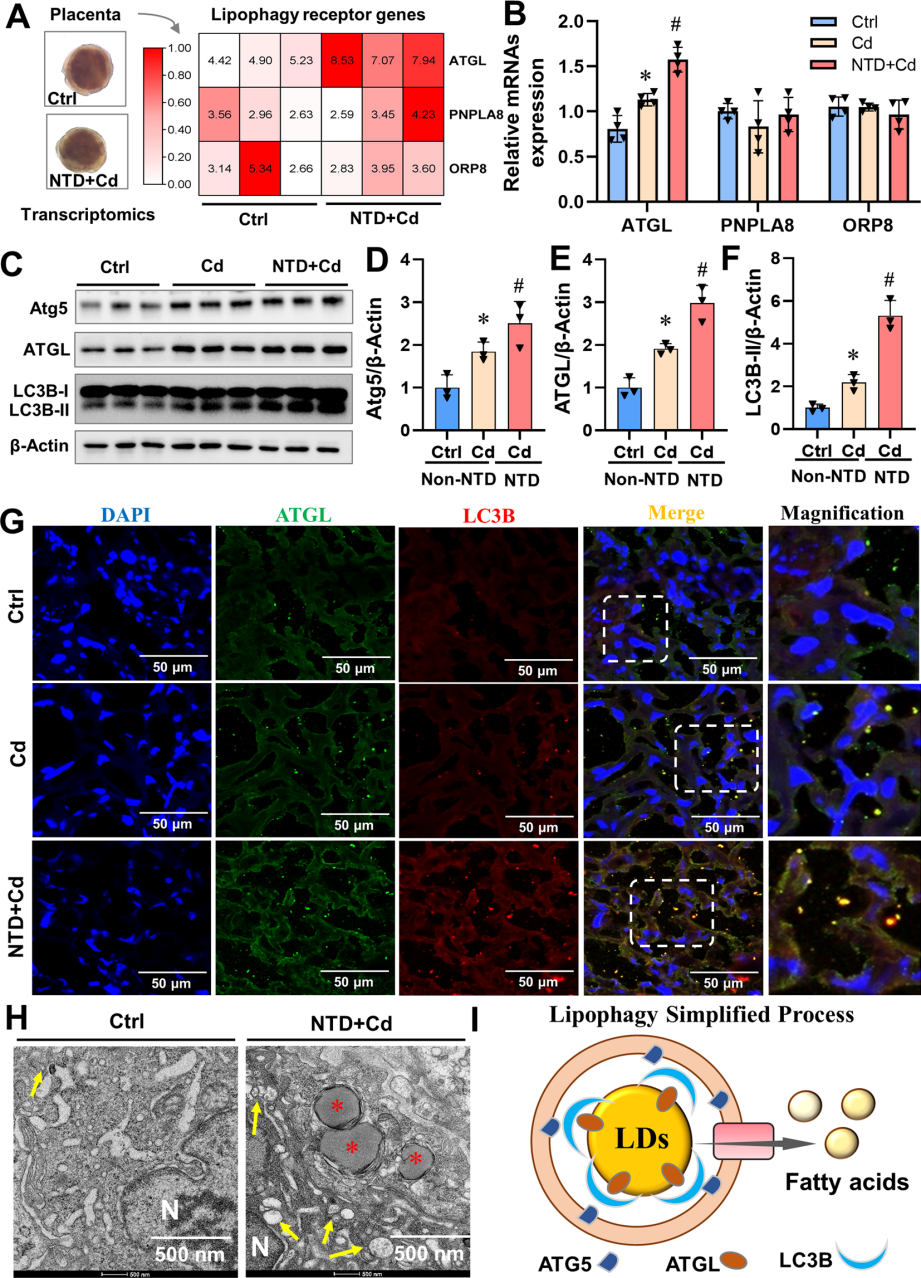
Gestational Cd exposure activates placental lipophagy. Pregnant mice were given CdCl_2_ (2.0 mg/kg) on GD8 and placentas were collected on GD16. (**A**) RNA sequencing of placenta and heat map of mouse placenta lipophagy receptor genes (ATGL/PNPLA8/ORP8), each group contains 3 placentas. (**B**) Relative mRNA levels of lipohagy receptor genes (*ATGL/PNPLA8/ORP8*) in mouse placentas, each group containing 4 placentas. (**C**) Placental immunoblots of Atg5, ATGL, and LC3B proteins in mice (*n*=3). (**D**-**F**) Atg5, ATGL, and LC3B quantification. (**G**) Mouse placental immunofluorescence images. ATGL colocalizes with LC3B (white arrows). Nuclei were tagged with DAPI. Scale bar: 50 μm (*n*=3). (**H**) Transmission electron microscopy analysis of autophagosomes and autophagosomes containing LD. Autophagosomes are represented by yellow arrows, and those that contain LD are represented by red *(*n*=3). (**I**) Lipophagy simplified process. Data were shown as *mean ± SD*. ^*^ *P* < 0.05, ^**^P < 0.01 compared to Ctrl group, ^#^ *P* < 0.01, ^##^ *P* < 0.01 compared to Cd (Non-NTD) group

To investigate the effects of maternal Cd exposure on placental lipophagy, we collected placentas for further experiments. The expression of lipophagy receptor in mouse placentas was presented as a heatmap showed that Cd exposure greatly enhanced the expression of ATGL (a lipophagy receptor) in mouse placentas with NTDs (Fig. 3A). Further analysis revealed that Cd exposure significantly upregulated the expression of *ATGL* mRNA in NTDs mouse placentas (Fig. 3B). Additionally, gestational Cd exposure greatly upregulated the expression of lipophagy-related proteins (Atg5, LC3B-II, and ATGL) in NTDs mouse placentas (Figs. 3C-F). Co-localization analysis showed that Cd exposure resulted in increased co-localization of LC3B with ATGL and ATGL with LDs in NTDs mouse placentas (Fig. 3G, Fig. S2A and, Fig. S2C and D). As showed in Fig. 3H and Fig. S2B, an increased number of LD-containing autophagosomes in Cd-treated NTDs mouse placentas was observed under transmission electron microscopy. Lipophagy, is a selectively degrades LDs, it is characterized by a spherical double membrane structure known as an LD-containing autophagosome (Fig. 3I). These findings suggest that maternal exposure to Cd activate placental lipophagy. Therefore, we conducted in vivo intervention experiments targeting placental lipophagy to clarify the role of lipohagy in Cd-induced NTDs.

### Inhibition of lipophagy aggravates Cd-evoked NTDs

**Fig. 4:**
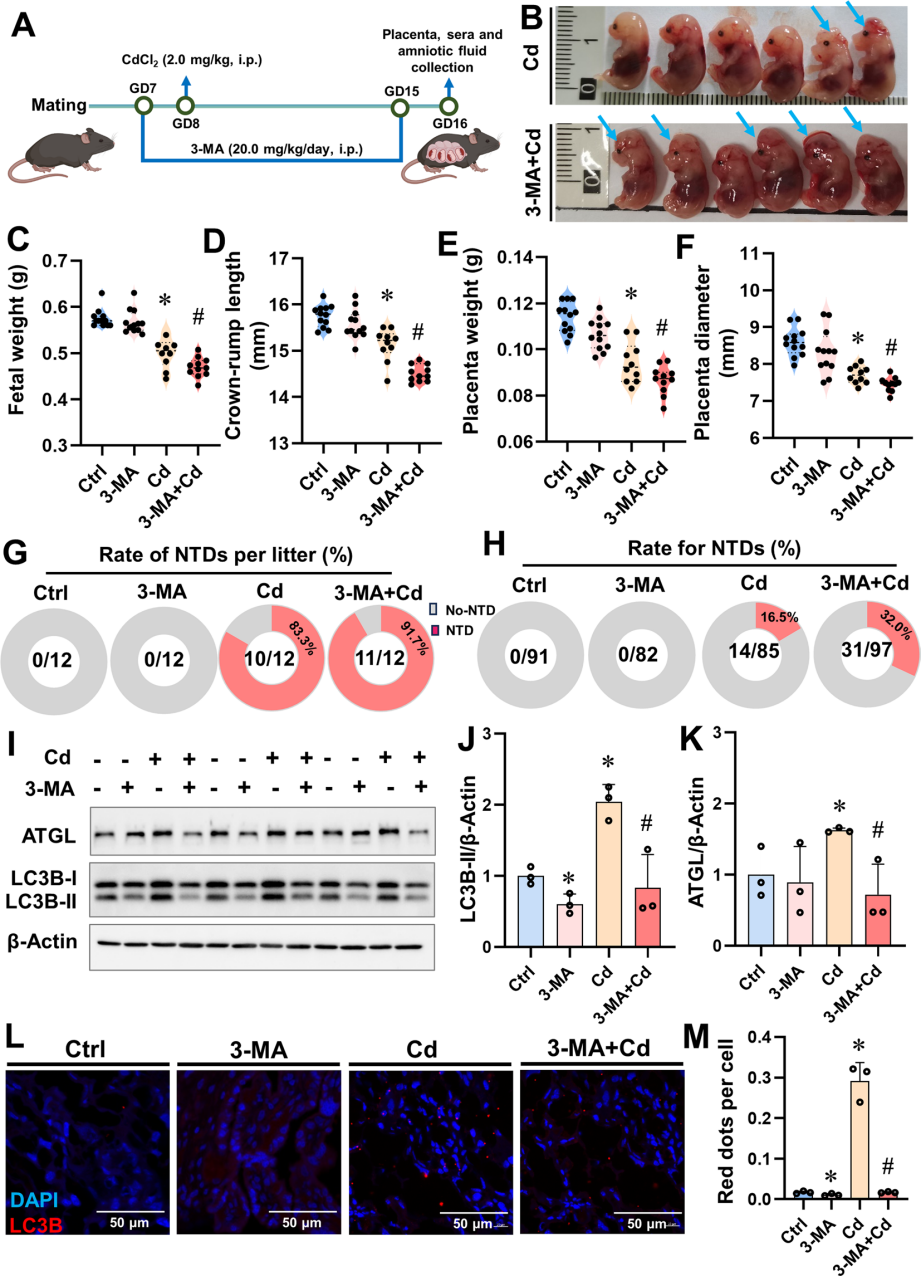
Inhibition of lipophagy aggravates Cd-evoked NTDs. Pregnant mice were pre-treated with 3-MA before Cd (2.0 mg/kg, i.p.) administration, placentae of Cd and 3-MA + Cd groups were from fetuses with NTDs, each group had 12 pregnant mice. (**A**) Research design of pregnant mice was pre-treated with 3-MA before Cd administration; (**B**) Representative image of fetus in Cd and 3-MA+Cd group. The blue arrows represent fetus with NTDs. (**C**) Fetal weight. (**D**) Crown-rump length. (**E**) Placenta weight. (**F**) Placenta diameter. (**G**) Litters with NTDs. (**H**) Rate for NTDs. (**I**) LC3B-II and ATGL immunoblots from mouse placentas (*n*=3). (**J** and **K**) LC3B-II and ATGL quantification. (**L**) Images of mouse placentas immunofluorescently stained. Nuclei were tagged with DAPI. Scale bar: 50 μm (*n*=3). (**M**) Red dots per cell. Data were shown as *mean ± SD*. * *P* < 0.05, **P < 0.01 *vs*. Ctrl mice. ^#^ *P* < 0.05, ^##^ *P* < 0.01 *vs*. Cd mice

To investigate the impact of lipophagy on Cd-evoked NTDs, mice were pre-treated with 3-MA (a lipophagy inhibitor) prior to Cd administration (Fig. 4A). As presented in Fig. 4B, maternal 3-MA pretreatment exacerbates Cd-induced fetal malformations. Pre-treatment with 3-MA markedly aggravated Cd-induced reduction of NTDs fetal weight and crown-rump length, as well as NTDs placentas weight and diameter (Figs. 4C-F). In the 3-MA+Cd groups, NTDs occurred in 32.0% (31/97) of fetuses and 91.7% (11/12) of litters were with NTDs (Figs. 4G and H). Additionally, we demonstrated that pre-treatment with 3-MA markedly reduced the levels of LC3B-II and ATGL proteins in Cd-treated NTDs mouse placentas (Figs. 4I-K), and the number of LC3B red fluorescence puncta in in 3-MA+Cd-treated NTDs mouse placentas were significantly lower than those in the Cd-treated group (Figs. 4L-M). In summary, inhibition of placental lipophagy aggravates Cd-induced NTDs. Therefore, the process of placental lipophagy is inhibited, leading to the development of more severe embryonic NTDs. This suggests that lipophagy may be a therapeutic target for Cd-induced NTDs.

### Loss of lipophagy aggravates Cd-evoked NTDs

**Fig 5:**
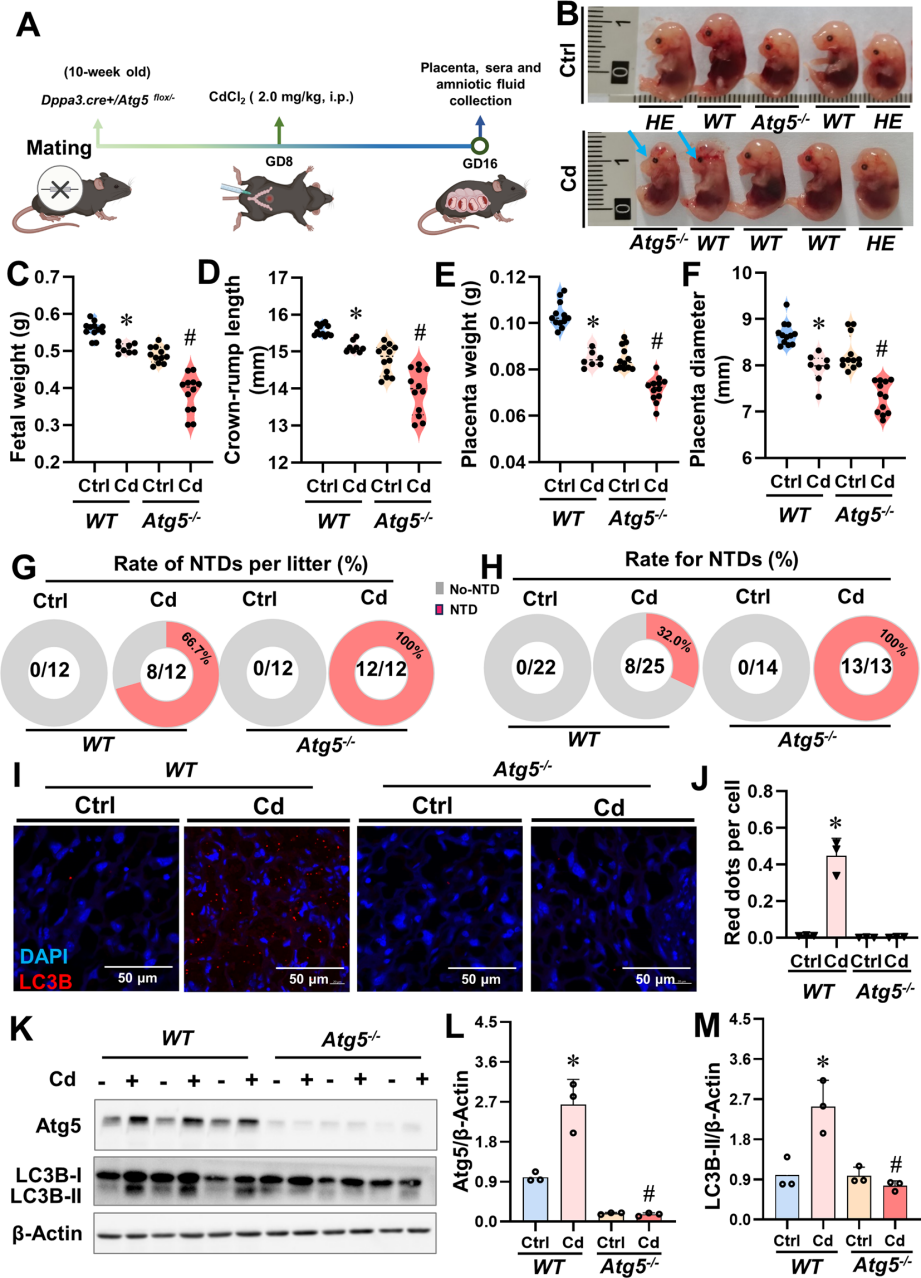
Loss of placental lipophagy aggravates Cd-evoked NTDs. *Dpp3-Cre/Atg5*^*flox/-*^ mice were treated with Cd (2.0 mg/kg, i.p.) on GD8, each group included 12 pregnant mice. (**A**) Construction process of placenta *Atg5* knockout mouse model. (**B**) Representative image of fetus in Cd and Ctrl group, the blue arrows represent fetus with NTDs, *WT* represents the wild type and *HE* represents the heterozygote. (**C**) Weight of fetal. (**D**) Crown-rump length. (**E**) Weight of placental. (**F**) Diameter of placental. (**G**) Litters with NTDs. (**H**) Rate for NTDs. (**I**) Images of mouse placentas immunofluorescently stained. Nuclei were tagged with DAPI. Scale bar: 50 μm (*n*=3). (**J**) Red dots per cell. (**K**) LC3B-II and Atg5 immunoblots from mouse placentas (*n*=3). (**L** and **M**) LC3B and Atg5 quantification. Data were shown as *mean ± SD*. * *P* < 0.05, **P < 0.01 *vs*. Ctrl mice. ^#^ *P* < 0.05, ^##^ *P* < 0.01 *vs*. Cd mice

In order to further strengthen the validation that inhibition of lipophagy aggravates Cd-induced NTDs, *Dpp3-Cre/Atg5*^*flox/-*^ mice (*Atg5* is a lipophagy-related gene) were exposed to Cd on GD8 (Figs 5A). Following Cd treatment, a significantly higher incidence of NTDs was observed in *Atg5*^*-/-*^ fetuses compared to their wild-type counterparts (Fig. 5B). Notably, *Atg5*^*-/-*^ fetuses with NTDs displayed significant reductions in fetal weight, crown-rump length, placental weight, and diameter following Cd exposure when compared to WT fetuses with NTDs (Figs. 5C-F). Remarkably, all litters from the *Atg5*^*-/-*^+Cd groups exhibited NTDs (12/12) (Fig. 5G and H). Furthermore, immunoblot analysis revealed a marked decrease in the levels of Atg5 and LC3B-II proteins as well as a reduction in the number of LC3B red puncta within Cd-treated NTD placentas from *Atg5*^*-/-*^ fetuses compared to those from WT fetuses (Figs. 5I-M). Above all, loss of lipophagy exacerbates Cd-induced NTDs, confirming the results of inhibition of lipophagy by the above drugs, and it was found that ATG5 gene deletion was very sensitive to Cd-induced NTD.

### Activation of lipophagy alleviates Cd-evoked NTDs

**Fig. 6:**
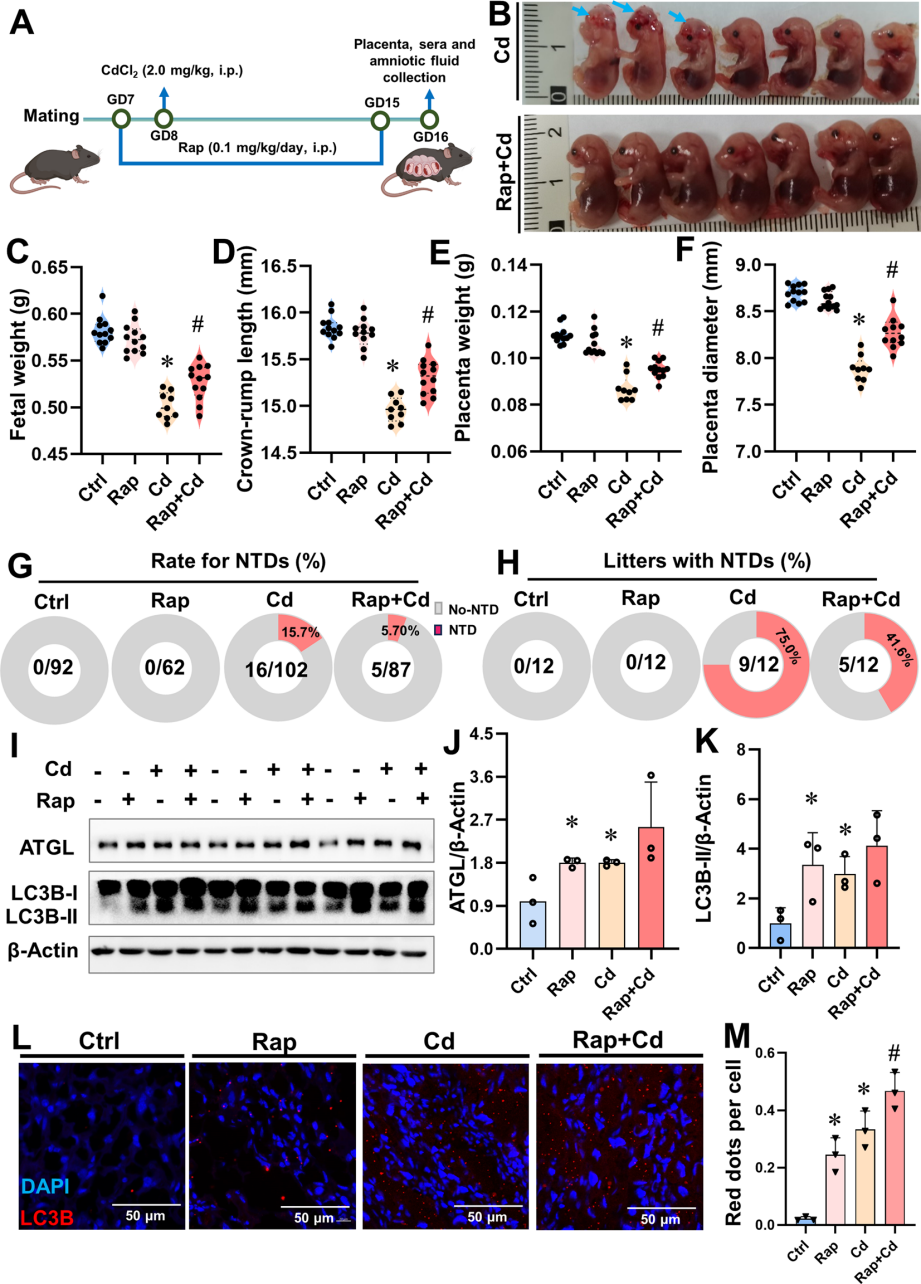
Activation of lipophagy alleviates Cd-evoked NTDs. Pregnant mice were pretreated with Rap before Cd (2.0 mg/kg, i.p.) administration, placentas of Cd groups were from fetuses with NTDs, each group included 12 pregnant mice. (**A**) Research design of pregnant mice was pretreated with Rap before Cd administration; (**B**) Representative image of fetus mice in Cd group and Rap+Cd group, the blue arrows represent fetus with NTDs; (**C**) Fetal weight(*n*=12). (**D**) Crown-rump length(*n*=12). (**E**) Placenta weight(*n*=12). (**F**) Placenta diameter(*n*=12). (**G**) Rate for NTDs. (**H**) Litters with NTDs. (**I**) Representative immunoblots of ATGL and LC3B-II proteins (*n*=3). (**J** and **K**) Quantification for ATGL and LC3B-II. (**L**) Images of mouse placentas immunofluorescently stained. Nuclei were tagged with DAPI. Scale bar: 50 μm, (*n*=3). (**M**) Red dots per cell. Data were shown as *mean ± SD*. * *P* < 0.05, **P < 0.01 *vs*. Ctrl mice. ^#^ *P* < 0.05, ^##^ *P* < 0.01 *vs*. Cd mice

To further elucidate the influence of lipophagy against Cd-triggered NTDs, mice were pre-treated with Rap (an activator of lipophagy) prior to Cd exposure (Figs. 6A). As shown in Fig. 6B, maternal Rap pretreatment significantly alleviates Cd-induced fetal malformations. Pre-treatment with Rap markedly alleviated Cd-evoked reduction of NTDs fetal weight and crown-rump length, as well as NTDs placentas weight and diameter (Figs. 6C-F). In the Rap+Cd groups, the incidence rate of NTDs was reduced to 5.70% (5/87), while 41.6% (5/12) of litters still exhibited NTDs (Figs. 6G and H). Furthermore, we demonstrated that pre-treatment with Rap increased levels of ATGL and LC3B-II proteins in Cd-treated NTDs mouse placentas (Figs. 6I-K and Figs. S3 A-C), and the number of LC3B red fluorescence puncta in Rap+Cd groups NTDs mouse placentas were significantly higher than those in the Cd-treated group (Figs. 6L and M). The above results found that activation of lipophagy alleviates Cd-induced NTDs, suggested that Rap may serve as a potential therapeutic agent for the treatment of NTDs.

### Placental lipophagy activation reverses Cd-induced the increase of LDL-C levels

**Fig. 7:**
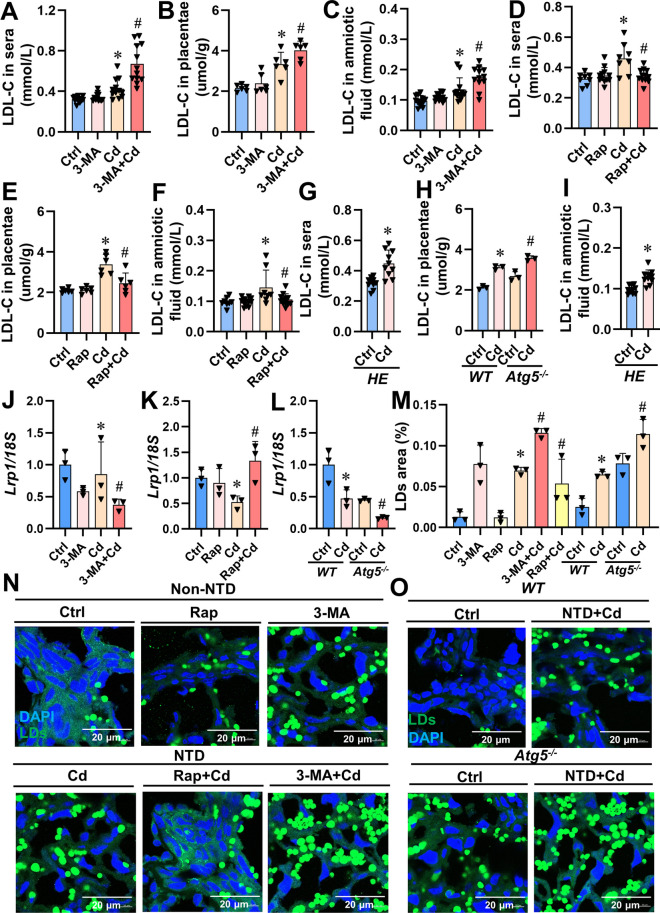
Placental lipophagy activation reverses Cd-induced the increase of LDL-C levels. Placentas of Cd and 3-MA+Cd groups were from fetuses with NTDs. (**A**-**F**) The level of LDL-C in maternal sera/placentas/amniotic fluid (*n*=6-12). (**G**) LDL-C in sera, *HE* represents *Dpp3-Cre/Atg5*^*flox/-*^ mice. (**H**) LDL-C in placentas. (I) LDL-C in amniotic fluid. (**J**-**M**) The level of LDL-C in maternal sera and amniotic fluid. (**N** and **O**) Placental staining with Bodipy 493/503. Scale bar: 20 μm, *n* = 3. Data were shown as *mean ± SD*. * *P* < 0.05, **P < 0.01 *vs*. Ctrl mice. ^#^ *P* < 0.05, ^##^ *P* < 0.01 *vs*. Cd mice

To further elucidate the association lipophagy and LDL-C, we conducted an analysis to determine the content of LDL-C. The results demonstrated that pre-treatment with 3-MA exacerbated the Cd-evoked elevation of LDL-C levels in maternal sera/ placentas/amniotic fluid (Figs. 7A-C), while pre-treatment with Rap significantly reversed this effect (Figs. 7D-F). Moreover, *Dpp3-Cre/Atg5*^*flox/-*^ pregnant mice exhibited a significant increase in LDL-C content in both amniotic fluid and maternal sera after Cd exposure (Figs. 7G and H), particularly evident in the higher levels of placentas LDL-C observed in *Atg5*^*-/-*^ fetuses compared to WT fetuses following Cd exposure (Fig. 7I). Pretreatment with 3-MA decreased *Lrp1* mRNA levels in Cd-treated NTDs placentas (Fig. 7J), whereas pretreatment with Rap increased *Lrp1* mRNA levels under similar conditions (Fig. 7K). Notably, the level of Lrp1 mRNA was markedly decreased in the Cd-treated NTDs placentas of *Atg5*^*-/-*^ fetuses relative to *WT* fetuses (Fig. 7L). Furthermore, Bodipy staining revealed that pretreatments with either 3-MA or Rap resulted respectively in an increase or decrease of lipid droplets (LDs) numbers prior to Cd treatment within mouse NTDs placentas samples (Figs. 7M and N), additionally, compared to those from *WT* fetuses, placental tissues from *Atg5*^*-/-*^ fetuses showed greater accumulation of LDs within mouse NTDs placentas samples examined by Bodipy staining methods (Figs. 7M and O). In conclusion, activation of lipophagy can mitigate increases induced by Cd exposure on LDL-C concentrations within mouse NTD placentas.

## Discussion

Worldwide, 1 in every 1000 pregnancies has a NTD and they cause enormous clinical, economic, and societal costs (Fimia et al. 2007; Zhang et al. 2016). Since China implemented a nationwide folic acid supplementation program in 2009, the prevalence of NTDs has dropped from 7.8% to 3.1% in five years (Chen et al. 2021a). Although many studies have confirmed that strengthening folic acid intake during perinatal pregnancy can reduce the incidence and recurrence rate of NTDs, not all types of NTDs can be prevented, so in-depth research into the causes of the disease is important to advance its diagnosis, treatment and research (Cao et al. 2024). Current research suggests that NTDs in humans follow a multifactor threshold model, in which a single factor may not be sufficient to disrupt normal neural tube closure, but rather a complex interaction between multiple genetic factors and environmental factors regulates the incidence and severity of the disease (Liang et al. 2023). Cadmium (Cd), a highly toxic environmental pollutant, is classified as a human carcinogen (Hong et al. 2021). Several epidemiologic studies have linked Cd contents to NTD risks (Chen et al. 2021b; Cherukad et al. 2012). During prenatal Cd exposure, CD-1 mice develop ectrodactyly in the forelimbs and tail deformities (Zhang et al. 2016). In this study, we found that gestational Cd (2.0 mg/kg, i.p.) exposure caused NTDs in C57BL/6J mice. Surprisingly, we also found that Cd exposure significantly lowered fetal weight, especially with NTDs embryos lower than normal embryos. The above results indicate that maternal Cd exposure leads to NTDs.

LDL-C levels increase during pregnancy, which may cause severe complications for both mothers and children (Pecks et al. 2016). The uptake of LDL by the placenta is well described and particularly involves the LDL-receptors, the reduced number of LDLRs causes LDL turnover rates to slow down and LDL-C levels to rise (Pecks et al. 2016). In this study, we found that transcriptome analysis of the NTDs mouse placentas showed that Cd exposure significantly down-regulation the expression of *Lrp1*, the genes were associated with positive regulation of LDL-C transport and axon development. Correspondingly, we found that Cd exposure significantly elevated the LDL-C levels in mouse NTDs placentas. Deficient Lrp2 mice have a dilated dorsal neural tube and cranial neural tube closure defects (Kowalczyk et al. 2021). Variants of Lrp2 in humans with NTDs, resulting in anencephaly and myelomeningocele, and found that folic acid supplementation alone did not help (Kur et al. 2014; Qiu et al. 2020). We then detected that the expression of folate transporter Pcft in NTDs mouse placenta did not decrease, folic acid levels in serum and amniotic fluid of NTDs mice caused by Cd exposure during pregnancy were unchanged (Fig. S4). The findings of this study implicate the pivotal role of the *Lrp1* gene in mouse susceptibility to NTDs, which could significantly contribute to a better understanding of their pathophysiology and facilitate therapeutic interventions for NTD prevention. LDL-C may be as a risk factor for NTDs for further analysis.

Lipid droplets (LDs) are dynamic lipid storage organelles that can be degraded by autophagy mechanisms to release neutral lipids, a process called lipophagy (Haidar et al. 2021). During neural tube closure, significant enhancement of autophagy was observed in the neural epithelial tissue (Huang et al. 2021; Wang et al. 2017). The latest research has found that maternal diabetes can suppress the autophagy function of neural epithelial cells, leading to NTDs; restoring the autophagy activity can reduce the occurrence of these situations (Ye et al. 2020). Previous study has found that lipophagy was activated when exposed to Cd in mouse placentas (Zhang et al. 2023). I In the study, our study also demonstrated that lipophagy was induced in Cd-treated NTDs placentas by increasing number of LD-containing autophagosomes, expression of lipophagy-related proteins. However, the precise mechanisms underlying placental lipophagy in Cd-induced NTDs remain poorly elucidated. By pharmacological and genetic means, we proved it for the first time that activation of placental lipophagy alleviated Cd-induced NTDs. The findings suggested that, in addition to folic acid, may serve as a potential therapeutic agent for the treatment of NTDs. Furthermore, targeting lipophagy could be a promising approach for managing NTDs.

LDL-C is formed by the combination of low-density lipoprotein and cholesterol (Lan et al. 2023). There is evidence that inhibited lipophagy leads to LD accumulation, whereas activated lipophagy degrades LDs (Maan et al. 2018; Zhang et al. 2023). Previous studies have shown that lipophagy alleviates Cd-caused LDs accumulation in placental and hepatocytes (Liu et al. 2021; Zhang et al. 2023). In this study, we present novel evidence demonstrating that lipophagy mitigates the Cd-evoked increase in LDL-C levels in NTDs mouse placentas. We found that there is less literature on the relationship between prenatal exposure to Cd and NTDs, and higher quality studies are needed. Future studies can use a multi-omics approach to further explore the placenta etiological mechanism of NTDs induced by environmental heavy metal exposure in key time windows such as neural tube closure.

## Conclusion

**Fig. 8:**
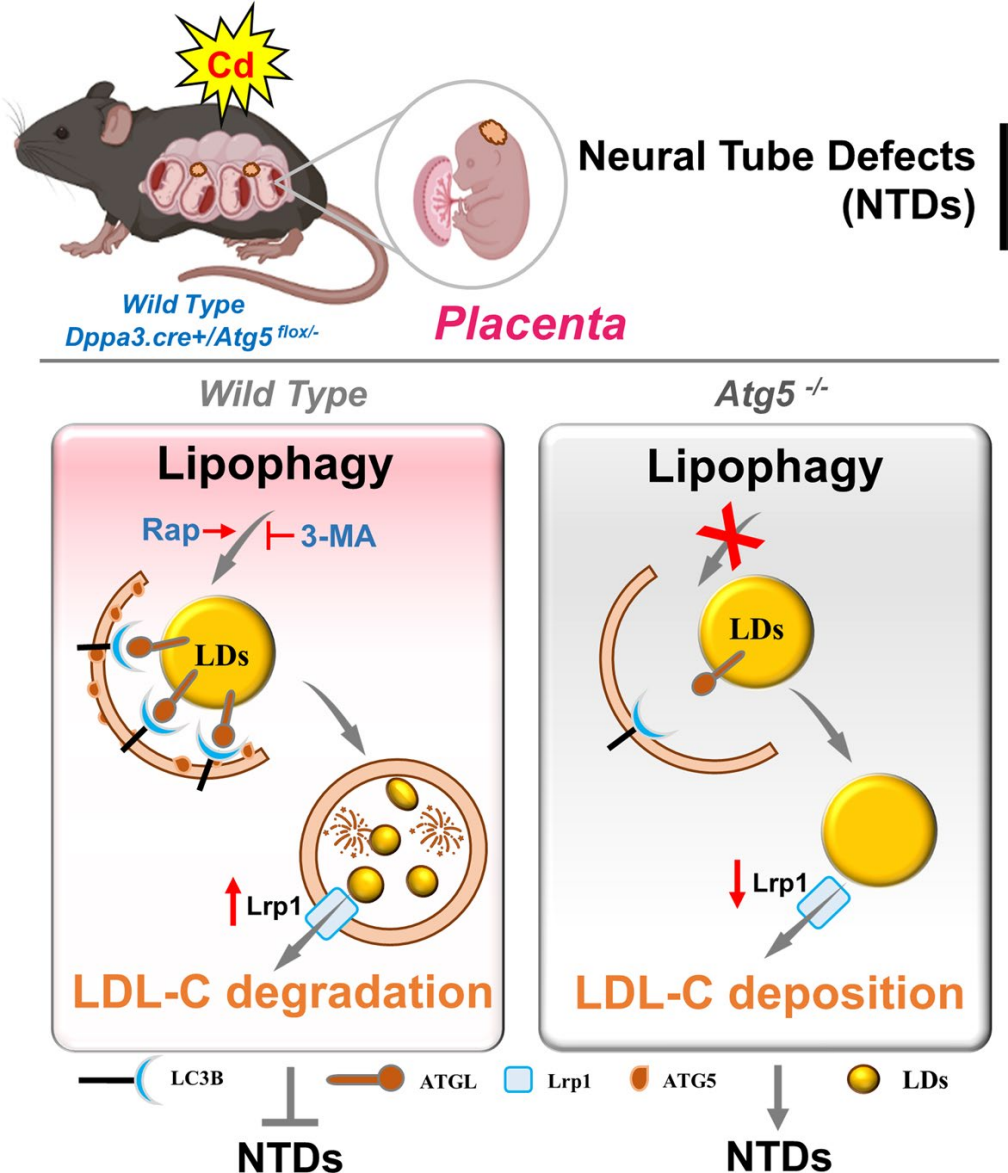
Activation of lipophagy ameliorates Cd-induced NTDs via reducing low density lipoprotein cholesterol levels in mouse placentas

This study presents novel findings demonstrating that activation of lipophagy mitigates gestational Cd-induced NTDs through reduction of LDL-C levels in mouse placentae (Fig 8). Implicate the pivotal role of the Lrp1 gene in mouse susceptibility to NTDs and LDL-C may be as a risk factor for NTDs for further analysis. By approaching disease treatment and prevention from a proactive standpoint, this study unveils the pivotal role of lipophagy in NTD treatment, thereby establishing a solid foundation for future investigations into lipophagy activation as a means to attenuate NTDs.

## Supplementary information

ESM 1 (DOCX 2409 kb)

## Data Availability

No datasets were generated or analysed during the current study.
