# Correction to: Activation of lipophagy ameliorates cadmium‑induced neural tube defects via reducing low density lipoprotein cholesterol levels in mouse placentas

**DOI:** 10.1007/s10565-024-09942-w

**Published:** 2024-11-15

**Authors:** Yu‑Feng Zhang, Shuang Zhang, Qing Ling, Wei Chang, Lu‑Lu Tan, Jin Zhang, Yong‑Wei Xiong, Hua‑Long Zhu, Po Bian, Hua Wang

**Affiliations:** 1 Department of Toxicology, School of Public Health, Anhui Medical University, Hefei, China; 2 Key Laboratory of Environmental Toxicology of Anhui Higher Education Institutes, Hefei, China; 3 Teaching and Research Section of Nuclear Medicine, School of Basic Medical Sciences, Anhui Medical University, Hefei, China; 4 Key Laboratory of Population Health Across Life Cycle (Anhui Medical University), Ministry of Education of the People’s Republic of China, Hefei, China

**Correction to: Cell Biol Toxicol (2024) 40:35**

10.1007/s10565-024-09885-2

After the publication of this article, we would like to acknowledge several errors in the Figures. The violin plot in Figure 1I and J is placed upside down, representative picture of Ctrl group mice in Figure 1B is in appropriate, the histograms in Figure 7J display the data from the 3-MA and Cd groups in a reversed order. The immunofluorescence representative pictures chosen for the Cd group of Figure 4L, the 3-MA group of Figure 7N and the Cd (NTD) group of Figure 7O are inappropriate. After checking all raw figures data, we find all these mistakes do not change the description and conclusions of manuscript. Therefore, it is necessary to correct some of the Figures given in the article. The correct form is as follows:
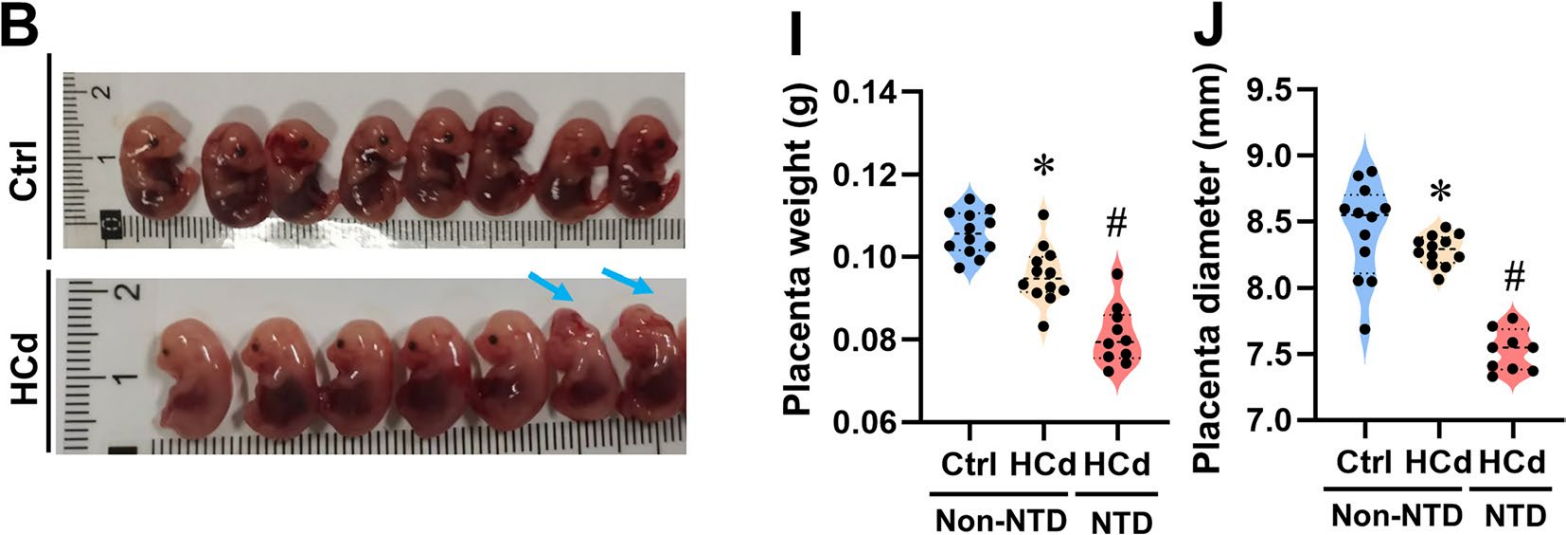


Figure 1. (B) Representative pictures of mice. (I) Placenta weight. (J) Placenta diameter.
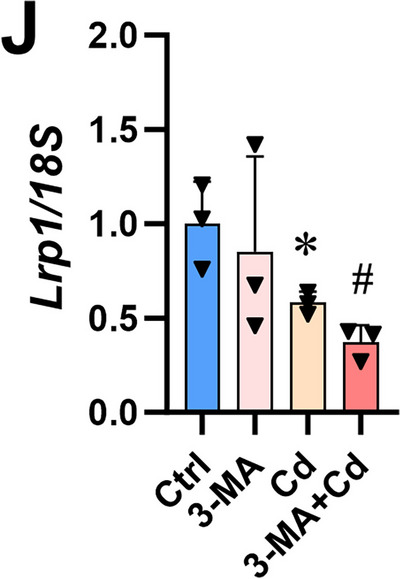


Figure 7. (J) *Lrp1/18S*.
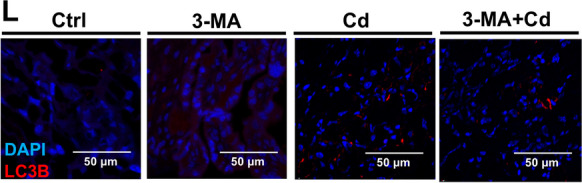


Figure 4. (L) Images of mouse placentas immunofluorescently stained.
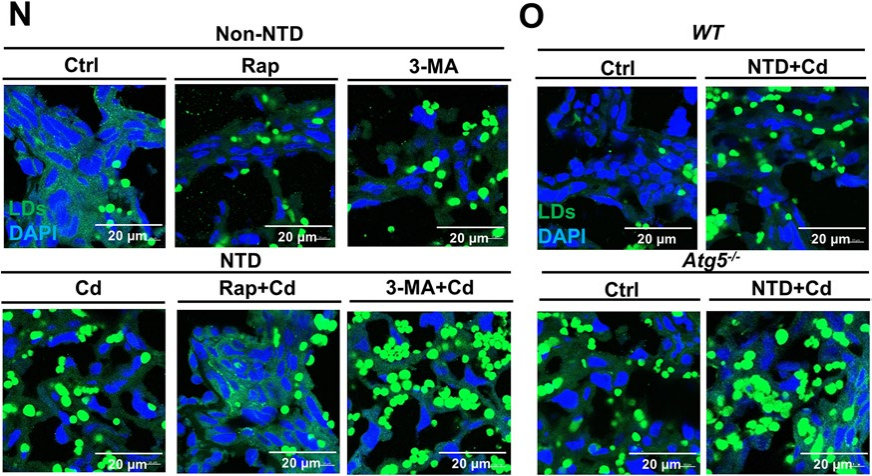


Figure 7. (N and O) Placental staining with Bodipy 493/503.

