# Supplementary material for: Activation of lipophagy ameliorates cadmium-induced neural tube defects via reducing low density lipoprotein cholesterol levels in mouse placentas
Source: Cell Biol Toxicol. 2024 May 21;40(1):35. doi: 10.1007/s10565-024-09885-2 (PMC11108957; doi:10.1007/s10565-024-09885-2)
Supplement: Supplementary file 1 — (DOCX 2409 kb) [file 10565_2024_9885_MOESM1_ESM.docx]

**Supplementary Material**

**Activation of Lipophagy Ameliorates Cadmium-induced Neural Tube Defects via Reducing Low Density Lipoprotein Cholesterol Levels in Mouse Placentas**

Yu-Feng Zhang ^a, b, d, 1^, Shuang Zhang ^a, b, 1^, Qing Ling ^a, b.^, Wei Chang ^a, b^, Lu-Lu Tan ^a, b^, Jin Zhang ^a, b^, Yong-Wei Xiong ^a, b^, Hua-Long Zhu ^a, b^, Po Bian ^d^ , Hua Wang ^a, b, c, *^

^a^ *Department of Toxicology, School of Public Health, Anhui Medical University, China*

**Affiliations:**

^b^ *Key Laboratory of Environmental Toxicology of Anhui Higher Education Institutes, China*

^c^ *Key Laboratory of Population Health Across Life Cycle (Anhui Medical University), Ministry of Education of the People’s Republic of China, China*

^d^ *Teaching and Research Section of Nuclear Medicine, School of Basic Medical Sciences, Anhui Medical University, China.*

*^1^ Equal contribution*

** To whom correspondence should be addressed to wanghuadev@ahmu.edu.cn*

**Corresponding authors:**

*Correspondence to: Department of Toxicology, School of Public Health, Anhui Medical University, Hefei 230032, China.

E-mail address: wanghuadev@ahmu.edu.cn (H. Wang),

Supplementary data **(Materials and methods) -** (Pages 2-11)

Supplementary data **(Fig. S1, Fig.S2, Fig.S2) -** (Pages 12-14)

Supplementary data **(Plagiarism Check report) -** (Pages 15)

**Supplementary data (Materials and methods)**

***1. Animals***

**Week age and origin of mice:**

We purchased 8-week-old wild-type C57BL/6J mice from Vital River (Beijing, China). Two weeks of adaptive feeding in SPF conditions were given to all mice. We purchased *Atg5^flox/flox^* C57BL/6 mice (NM-CKO-00131) and *Dpp3-Cre* C57BL/6 mice (NM-KI-00040) from Shanghai Nanmo Biotechnology Development Co., LTD. The detailed construction method of the gene mouse model was carried out on the basis of the previous study (Zhang et al., 2023).

**Basic living conditions of mice:**

We purchased 8-week-old wild-type C57BL/6J mice from Vital River (Beijing, China). Two weeks of adaptive feeding in SPF conditions were given to all mice. We purchased *Atg5^flox/flox^* C57BL/6 mice (NM-CKO-00131) and Dpp3-Cre C57BL/6 mice (NM-KI-00040) from Shanghai Nanmo Biotechnology Development Co., LTD. The mice were provided unrestricted access to food and water, while being housed in a specific pathogen-free (SPF) environment maintained at a constant temperature of 21 ℃ to 23 ℃ throughout the diurnal cycle. A regular light-dark schedule was implemented with automated lights turning on at 7 a.m. and promptly turning off at 7 p.m. The humidity in the rat room was maintained within a range of 55% to 65%.

**Animals matting:**

After two weeks of adaptive feeding, healthy two males and four females were mated in a cage at 8:30 p.m. The female mice underwent examination for vaginal plug at 7:00 a.m. the following day, with the identification of vaginal plug marking gestational day 0 (GD0). Body weight, diet, and water volume were recorded on the morning of GD0-GD16. Mating continued for a period of 3 to 5 days, and when the mating rate significantly declined, it was discontinued after an additional 2 days until the desired number of pregnant mice was achieved. Subsequently, pregnant mice were weighed and on GD7, they were categorized into groups based on ascending body weight order. Random allocation was then performed to assign pregnant mice to different drug treatment groups for administration and control. Pregnant mice treated with normal saline served as the control group.

**Cadmium dose selection basis:**

Cadmium is a highly prevalent toxic pollutant in the environment and has emerged as an urgent and recurring global challenge of paramount concern. The Cd dose was chosen according to the human internal exposure dose and on the basis of a large number of previous studies.

Based on a population study, the blood Cd concentration in the women who delivered babies with low birth weight (3.37±2.58 μg/L) were significantly higher than those babies with normal weight (2.22±1.99 μg/L) (Samejo et al., 2019). Our previous study found that maternal sera Cd concentration was 3.25±0.46 μg/L when pregnant mice were given with 4.5 mg/kg CdCl_2_, which was close to the blood Cd in the women who delivered babies with low birth weight (Wang et al., 2016b).

Meaningfully, the sera Cd concentration of Cd-treated pregnant mice was also close to the blood Cd concentration of current smokers in Pakistan (3.54±0.38 μg/L) and China (median=2.58, range from 0.95 to 5.36 μg/L) according to several recent human studies (Satarug et al., 2010), and lower than the standard trigger level of Cd (5 μg/L) for medical surveillance in Occupational Safety & Health Administration, USA (Ikeh-Tawari et al., 2013). CD-1 pregnant mic exposure to CdCl_2_ (2.5 mg/kg and 5.0 mg/kg) led to the occurrence of NTDs (Zhang et al., 2016). In a previous study conducted by our team, we observed detectable levels of Cd in the serum (7.50±0.84 mg/L) and testis (0.23±0.02 mg/g) of C57BL/6 male mice following treatment with CdCl_2_ (2.00 mg/kg) (Zhou et al., 2021). These concentrations closely resemble those found in humans residing in areas with high Cd contamination or individuals who smoke tobacco products. As above, the dose of Cd was selected as 1.0 mg/kg and 2.0 mg/kg in this study.

In our previous studies, we established acute and subchronic murine models of Cd-impaired fetal and placental development. In the acute mouse model, the pregnant mice were given with a single high-dose CdCl_2_ (2.5 or 5.0 mg/kg) via intraperitoneal injection (i.p.) (Wang et al., 2016). In the subchronic mouse model, the pregnant mice were exposed to repetitive low-dose CdCl_2_ (150 mg/L) through drinking water (Xiong et al., 2020). CD-1 pregnant mic exposure to CdCl_2_ (2.5 mg/kg and 5.0 mg/kg) led to the occurrence of NTDs (Zhang et al., 2016). To explore the detailed pathogenic mechanism of placenta for Cd-induce NTDs, the present study chose the i.p. treatment.

**Animal experiment design:**

Experimental design 1: To establish a model of NTDs caused by Cd exposure during pregnancy. The pregnant mice were divided into Control (Ctrl) group, Low Cadmium group (LCd) and High Cadmium group (HCd), with 12 mice in each group. On GD8, the mice in the Ctrl group were intraperitoneally injected with normal saline, and the mice in the LCd and the HCd group were intraperitoneally injected with CdCl_2_ (1.0 and 2.0 mg/kg), respectively. At the same time, electron microscope sections were prepared to observe the autophagosomes in placental trophoblast cells.

Experimental design 2: To investigate the role of lipophagy in Cd-induced NTDs. 3-methyladenine (3-MA), an inhibitor of lipophagy, and rapamycin (Rap), an activator of lipophagy, were used for intervention experiments. The pregnant mice were divided into Ctrl group, Cd group, 3-MA group and 3-MA + Cd group, with 12 pregnant mice in each group. On GD8, the pregnant mice in Cd group and 3-MA + Cd group received CdCl_2_ (2.0 mg/kg) by intraperitoneal injection. Pregnant mice in 3-MA group and 3-MA + Cd group received daily intraperitoneal injection of 3-MA (20 mg/kg/day) from GD7 to GD15 (Liu et al., 2019). At the same time, pregnant mice were divided into Ctrl group, Cd group, Rap group and Rap+Cd group, with 12 pregnant mice in each experimental group. On GD8, the pregnant mice in Cd group and Rap + Cd group received CdCl_2_ (2.0 mg/kg) by intraperitoneal injection. Pregnant mice in Rap group and Rap + Cd group received daily intraperitoneal injection of Rap (0.1 mg/kg/day) from GD7 to GD15 (Dai et al., 2021).

Experimental design 3: The construction method of placenta *Atg5* knockout model is based on the previous research results of our research group (Zhang et al., 2023). The *Dpp3-Cre/Atg5^flox/-^* pregnant mice were divided into Ctrl group and Cd group, with 12 mice in each group. On GD8, the mice in the Ctrl group were intraperitoneally injected with normal saline, and the mice in the Cd group were intraperitoneally injected with CdCl_2_ (2.0 mg/kg).

The mice were euthanized on the morning of GD16, and samples of placenta, fetal mice, amniotic fluid, as well as maternal and fetal blood were collected. The placentas were collected based on the observed neural tube defects, with each placenta being individually isolated. In the Ctrl group and the experimental group exposed to Cd, the fetal mice were removed from the blood and debris, and then stored in 4% paraformaldehyde for sampling and dissection immediately to observe the neural tube defects. Maternal blood, amniotic fluid and fetal blood samples of pregnant mice were placed on ice without shaking, centrifuged, and stored at -80 ℃ for subsequent biochemical tests.

**Animal ethics:**

All mice were anesthetized by intraperitoneal injection of tribromoethanol (100 mg/kg, MKCM1080, Sigma) and then euthanized humanely. Accepted guidelines for humane outcomes were followed. The animal experiments involved in this study have been approved by the Animal Ethics Committee of Anhui Province and the Laboratory Animal Center of Anhui Medical University (approval number: LLSC20190297).

**Detailed record of the number of mouse samples in animal experiment**

Table S1: Detailed record of the number of mouse samples in animal experiment 1.

| Groups | Number of pregnant mice | Number of fetal mice | No-NTDs | NTDs | Total litters | No-NTDs litters | NTDs  litters |
| --- | --- | --- | --- | --- | --- | --- | --- |
| Ctrl | 12 | 93 | 93 | 0 | 12 | 12 | 0 |
| LCd | 12 | 90 | 90 | 0 | 12 | 12 | 0 |
| HCd | 12 | 93 | 77 | 16 | 12 | 2 | 10 |

Table S2: Detailed record of the number of mouse samples in animal experiment 2.

| Groups | Number of pregnant mice | Number of fetal mice | No-NTDs | NTDs | Total litters | No-NTDs litters | NTDs  litters |
| --- | --- | --- | --- | --- | --- | --- | --- |
| Ctrl | 12 | 91 | 91 | 0 | 12 | 12 | 0 |
| Cd | 12 | 85 | 71 | 14 | 12 | 2 | 10 |
| 3-MA | 12 | 82 | 82 | 0 | 12 | 12 | 0 |
| 3-MA+Cd | 12 | 97 | 60 | 37 | 12 | 1 | 11 |

Table S3: Detailed record of the number of mouse samples in animal experiment 2.

| Groups | Number of pregnant mice | Number of fetal mice | No-NTDs | NTDs | Total litters | No-NTDs litters | NTDs  litters |
| --- | --- | --- | --- | --- | --- | --- | --- |
| Ctrl | 12 | 92 | 92 | 0 | 12 | 12 | 0 |
| Cd | 12 | 102 | 86 | 16 | 12 | 3 | 9 |
| Rap | 12 | 90 | 90 | 16 | 12 | 12 | 0 |
| Rap+Cd | 12 | 87 | 82 | 5 | 12 | 7 | 5 |

Table S4: Detailed record of the number of mouse samples in animal experiment 3.

| Groups  *Dpp3-Cre/Atg5^flox/-^* | Number of pregnant mice | Number of fetal mice | No-NTDs | NTDs | Total litters | No-NTDs litters | NTDs  litters |
| --- | --- | --- | --- | --- | --- | --- | --- |
| Ctrl | 12 | 22(*Wt*) | 22 | 0 | 12 | 12 | 0 |
|  |  | 45(HE) | 45 | 0 |  |  |  |
|  |  | 14(*Atg5-/-*) | 14 | 0 |  |  |  |
| Cd | 12 | 25(*Wt*) | 17 | 8 | 12 | 0 | 12 |
|  |  | 47(HE) | 22 | 25 |  |  |  |
|  |  | 13(*Atg5-/-*) | 0 | 13 |  |  |  |

***2. Cd measurement***

The Cd content in the placenta was determined using graphite furnace atomic absorption spectrometry (GFAAS; model: TAS-990; Purkinje General Instrument Co., Ltd., Beijing, China). Mouse samples were prepared and analyzed following the previously described methodology (Ji et al., 2011). Moreover, 100 mg of mouse placentas were subjected to digestion and subsequent evaporation to dryness. The resulting residue was then reconstituted in a solution containing 1.0% HNO_3_, followed by detection using GFAAS. Each sample was examined in triplicate. The tubes were then stored at room temperature for 12 hours to obtain a clear and transparent digestive solution. After this period, the mixture was evaporated until almost dry and the residue was reconstituted with 1.0% HNO_3_. Subsequently, a 10 μl aliquot of the solution was extracted for analysis using atomic absorption spectrometry. Each sample underwent triplicate analyses to ensure accuracy and precision. The detection limit for Cd was determined to be 0.01 μg/L. To prevent any potential contamination from external sources of Cd, all polypropylene tubes and pipettes used were DNase and RNase free as well as sterile; furthermore, they were pre-treated by soaking in 10% ultrapure HNO_3_ at room temperature overnight followed by rinsing with deionized water.

***3. RNA sequencing and data analysis***

Total RNA was isolated using the Trizol Reagent (Invitrogen, China), after which the concentration, quality and integrity were determined using a NanoDrop spectrophotometer (Thermo Scientific). Three micrograms of RNA were used as input material for the RNA sample preparations. Sequencing libraries were generated according to the following steps. Firstly, mRNA was purified from total RNA using poly-T oligo-attached magnetic beads. Fragmentation was carried out using divalent cations under elevated temperature in an Illumina proprietary fragmentation buffer. First strand cDNA was synthesized using random oligonucleotides and Super Script II. Second strand cDNA synthesis was subsequently performed using DNA Polymerase I and RNase H. Remaining overhangs were converted into blunt ends via exonuclease/polymerase activities and the enzymes were removed. After adenylation of the 3′ ends of the DNA fragments, Illumina PE adapter oligonucleotides were ligated to prepare for hybridization. To select cDNA fragments of the preferred 400-500 bp in length, the library fragments were purified using the AMPure XP system (Beckman Coulter, Beverly, CA, USA). DNA fragments with ligated adaptor molecules on both ends were selectively enriched using Illumina PCR Primer Cocktail in a 15 cycle PCR reaction. Products were purified (AMPure XP system) and quantified using the Agilent high sensitivity DNA assay on a Bioanalyzer 2100 systemS7 (Agilent, USA). The sequencing library was then sequenced on NovaSeq 6000 platform (Illumina, USA) performed by Shanghai Personal Biotechnology Cp. Ltd (Shanghai, China). Samples were sequenced on the platform to get image files, which were transformed by the software of the sequencing platform, and the original data in FASTQ format (Raw Data) was generated. Sequencing data contains a number of connectors, low-quality Reads, so high quality sequence (Clean Data) were got by Cutadapt (v1.15) software to filter the sequencing data for further analysis. The reference genome and gene annotation files were downloaded from genome website. The filtered reads were mapping to the reference genome using HISAT2 v2.0.5. we used HTSeq (0.9.1) statistics to compare the Read Count values on each gene as the original expression of the gene, and then used FPKM to standardize the expression. Then difference expression of genes was analyzed by DESeq (1.30.0) with screened conditions as follows: expression difference multiple |log2FoldChange| > 1.2, significant P-value < 0.05. At the same time, we used R language heatmap (1.0.8) software package to perform bi-directional clustering analysis of all different genes of samples. We got heatmap according to the expression level of the same gene in different samples and the expression patterns of different genes in the same sample with Euclidean method to calculate the distance and Complete Linkage method to cluster. We mapped all the genes to Terms in the Gene Ontology database and calculated the numbers of differentially enriched genes in each Term. Using top GO to perform GO enrichment analysis on the differential genes, we calculated P-value byS8 hypergeometric distribution method (the standard of significant enrichment is P-value <0.05), and identified the GO term with significantly enriched differential genes to determine the main biological functions performed by differential genes. Cluster Profiler (3. 4. 4) software was used to carry out the enrichment analysis of the KEGG pathway of differential genes, focusing on the significant enrichment pathway with P-value <0.05.

***4. Immunoblotting***

In this study, one half of the placenta was used for immunoblot analysis, whereas the other half was used for RT-PCR. The placentas used in the experiments were from different pregnant mice, and each group of experiments contained 3 placentas. Total protein was extracted using RIPA buffer, RIPA buffer was supplemented with protease inhibitors (Protease Inhibitor Cocktail, Cat. No.: HY-K0010, MedChemExpress). To measure protein concentration, the Pierce BCA Protein Analysis Kit (Cat, No.:23225, Thermo Fisher Scientific). was used as directed by the manufacturer. Total placental lysates were separated electrophoretically by 12.5% SDS-PAGE and transferred to a PVDF membrane, then transferred to freshly prepared 5% skim milk and closed at room temperature for 1.5 hours. Following that, PVDF membranes were incubated for an hour to three hours with primary antibodies. A secondary antibody was incubated for 1-2 hours on PVDF membranes after washing. Lastly, gray-scale imaging of PVDF membrane was performed using Chemiluminescence reagents (Cat, No.:34094, Thermo Fisher Scientific) with a universal imaging system. Image J software was used to analyze the gray level of the corresponding bands and calculate the amount of the target protein.

**Total protein extraction:**

Total protein was extracted using RIPA buffer, RIPA buffer was supplemented with protease inhibitors (Cat. No.: HY-K0010, MedChemExpress), mix well and set aside on ice. After thawing the placenta samples preserved at -80 °C, they were dissected into two halves using scissors, weighed, and then transferred to 1.5 mL enzyme-free EP tubes followed by addition of corresponding amount of RIPA lysate. The prepared samples were homogenized using an electric homogenizer, and each sample was homogenized up and down for 5 seconds. After one set of samples was homogenized, the homogenizer drill was cleaned with pure water and the next set of samples was manipulated. After repeating the above procedure once, the mixture was allowed to stand on ice for 30 min, during which the upper and lower mixing was performed several times at approximately 5 min intervals. After the samples were completely set, they were placed into a centrifuge for cold storage (4 ° C) and centrifuged at a speed of 15000 g for 15 min. The supernatant was aspirated carefully to avoid suction into the bottom sediment and top layer of fat. The centrifugation step was repeated once, and finally the supernatant was carefully aspirated using the tip of the gun to obtain total protein.

**Total protein quantification:**

Materials required for the experiments included a BCA kit (Cat, No.:23225, Thermo Fisher Scientific), ultrapure water, and 96-well plates. The procedure was as follows: first, standard, blank, and sample Wells were added sequentially to a total volume of 20 µL. Then, 200 µL of BCA mixture (solution A: solution B =50:1) was added to each well. The 96-well plate was placed in a microplate reader for absorbance detection. The temperature was 37 ℃, the incubation time was 15 min, the vibration time was 5 s, and the detection wavelength was 562 nm. Then a curve was made according to the absorbance and concentration of the standard curve, and the absorbance of the sample was substituted into the curve and multiplied by the corresponding dilution to calculate the protein concentration. After quantification, the samples of each group were mixed with shaking and centrifuged, and the proteins were denatured by heating at 100 ° C for 10 min. After cooling, the total protein samples were stored at -20 ° C for later use.

**Protein immunoblotting:**

Total placental lysates were separated electrophoretically by 12.5% SDS-PAGE and transferred to a PVDF membrane. The membrane was washed in TBST buffer for 5 minutes, then transferred to freshly prepared 5% skim milk and closed at room temperature for 1.5 hours. After milk sealing, PVDF membranes were placed in the primary antibody to be tested (LC3B/Atg5/Atgl/Lrp1) and incubated at room temperature for 1 h to 3 h (depending on the expressed abundance of the target protein in the tissue). At the end of the incubation with the primary antibody, the membranes were washed three times for 8 min each in TBST buffer. After washing, the membranes were then placed in secondary antibodies of the same origin as the primary antibodies and incubated for 1 to 2 hours at room temperature. Subsequently, the membranes were placed in TBST buffer for three washes of 8 min each. Gray-scale imaging of PVDF membrane was performed using Chemiluminescence reagents (Cat, No.:34094, Thermo Fisher Scientific) with a universal imaging system. Image J software was used to analyze the gray level of the corresponding bands and calculate the amount of the target protein.

***5.*** ***Real-time qPCR***

The results of RNA agarose gel electrophoresis in supplemental material for the revised manuscript.


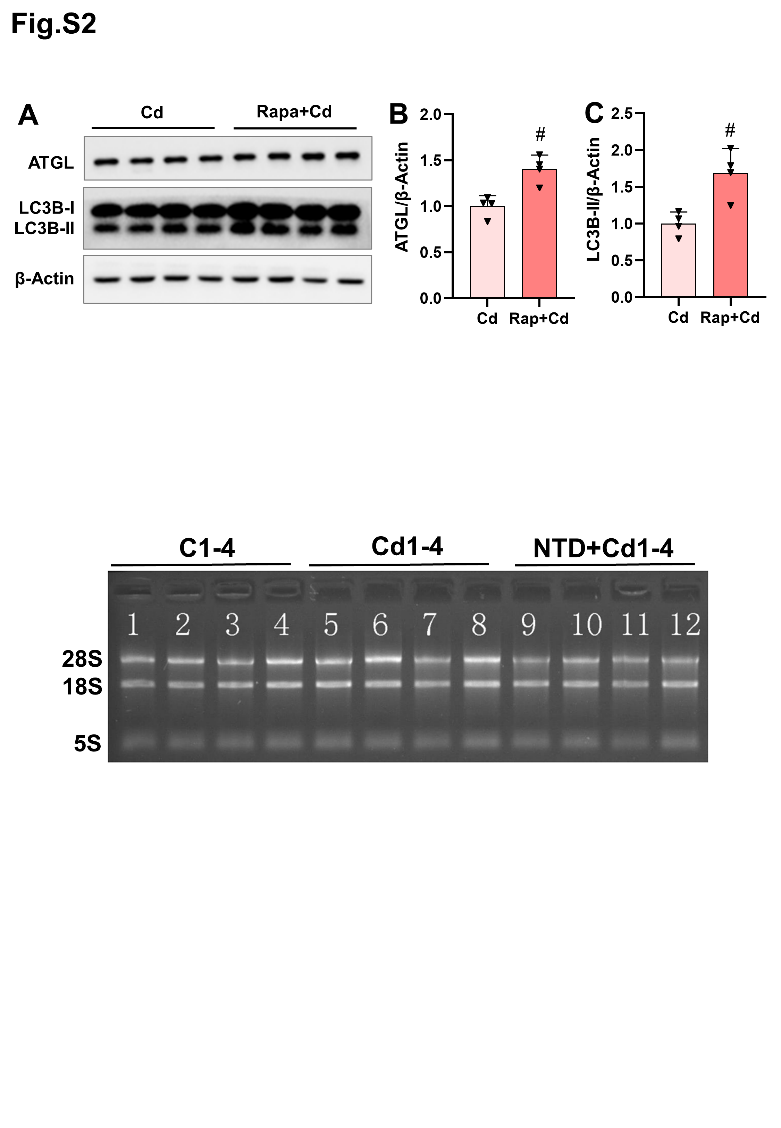
.

**Supplementary data (Fig. S1, Fig.S2,** **Fig.S2)**


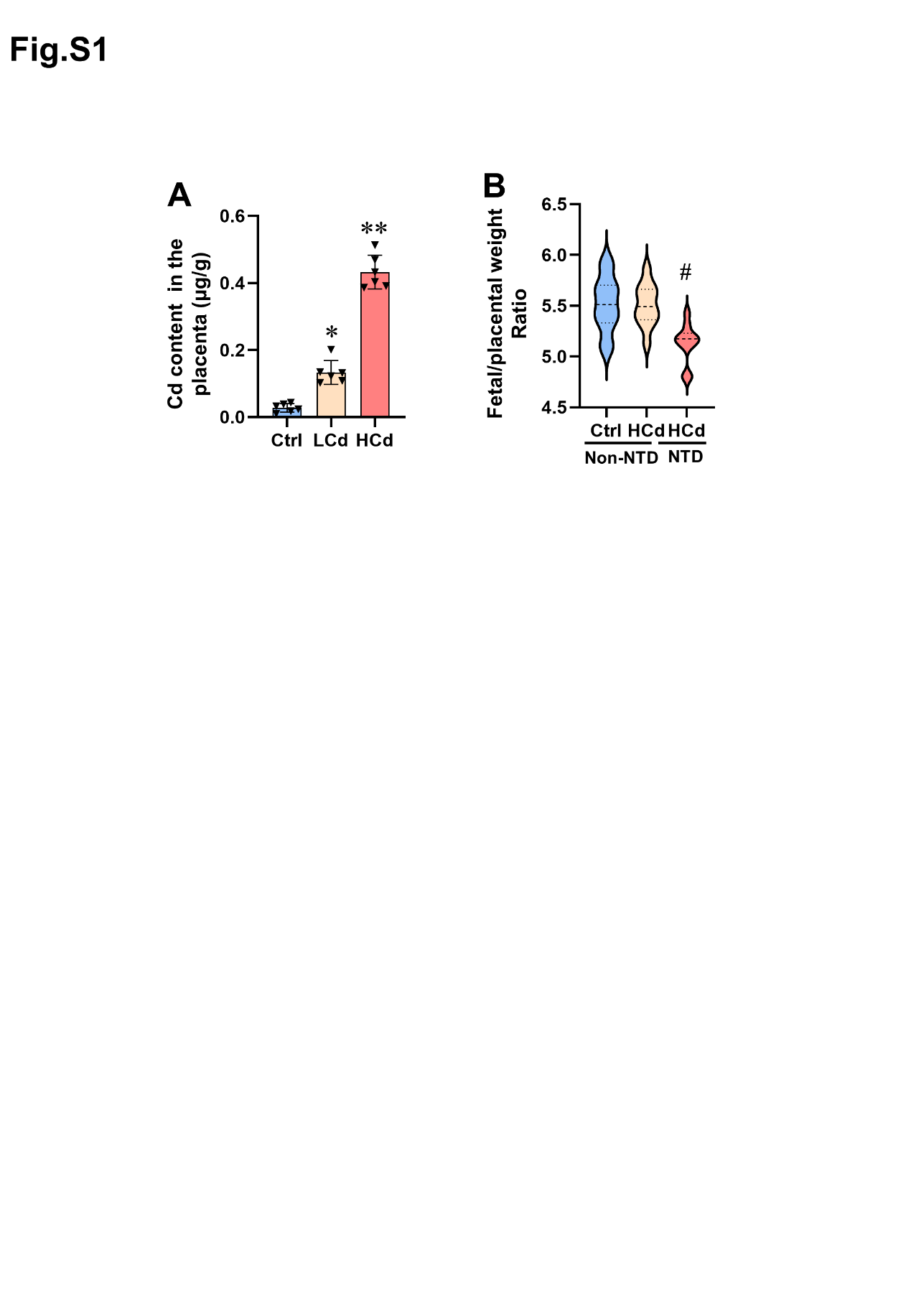


Figure S1A. (A) Cadmium content in the placenta (μg/g). (B) Fetal/placental weight Ratio.


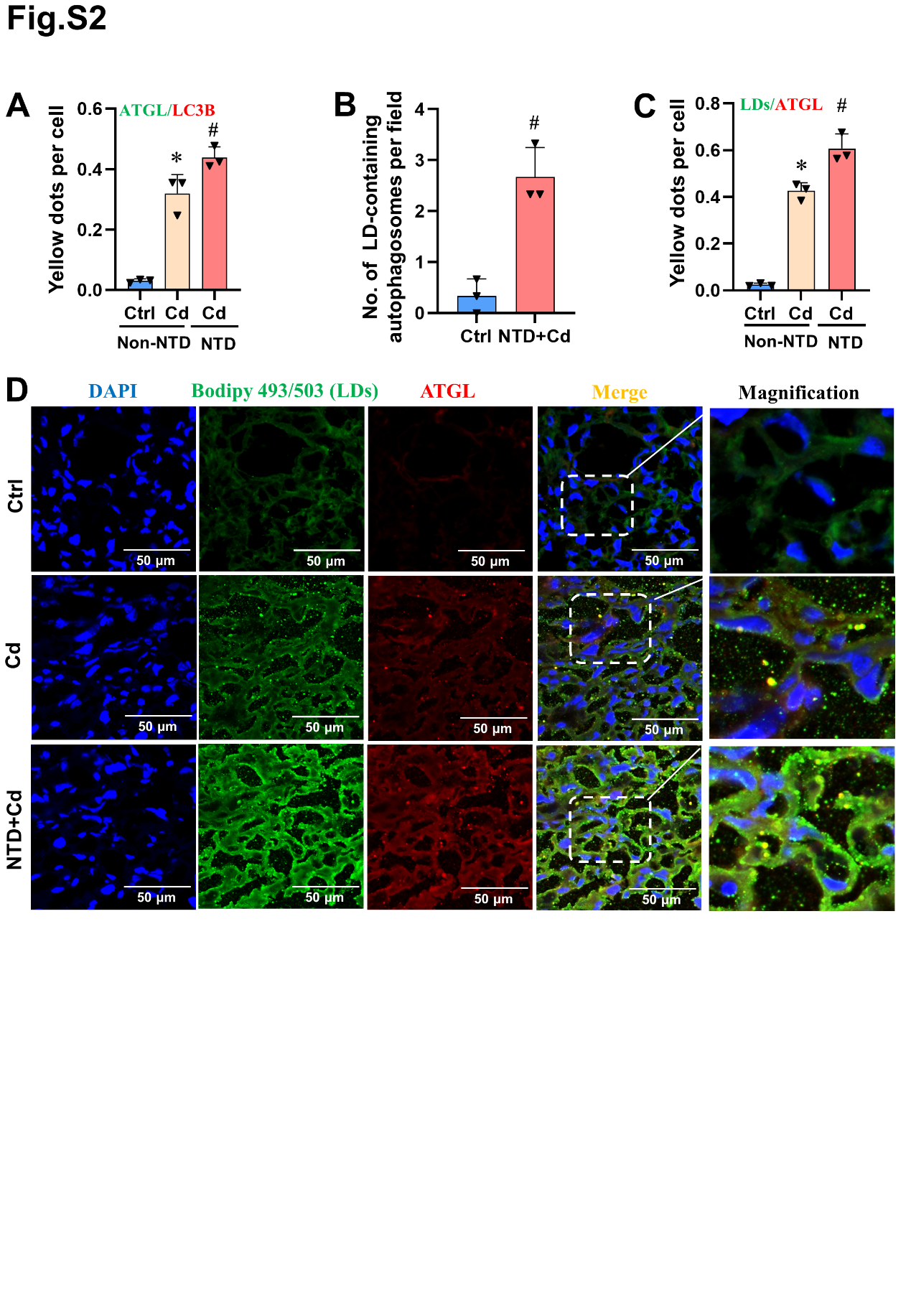


**Fig. S2. Gestational cadmium exposure activates placental lipophagy.** On GD16, mouse placentas were collected. (A) ATGL colocalizes with LC3B, yellow dots per cell. (B) No. of LD-containing autophagosomes per field. (C) ATGL colocalizes with LDs, yellow dots per cell. (D) Mouse placentas immunofluorescence images. ATGL colocalizes with LDs. Nuclei were tagged with DAPI. Scale bar: 50 μm. Data were shown as *mean ± SD*. ^*^ *P* < 0.05, ^**^*P* < 0.01 compared to Ctrl group, ^#^ *P* < 0.01, ^##^ *P* < 0.01 compared to Cd (Non-NTD) group.


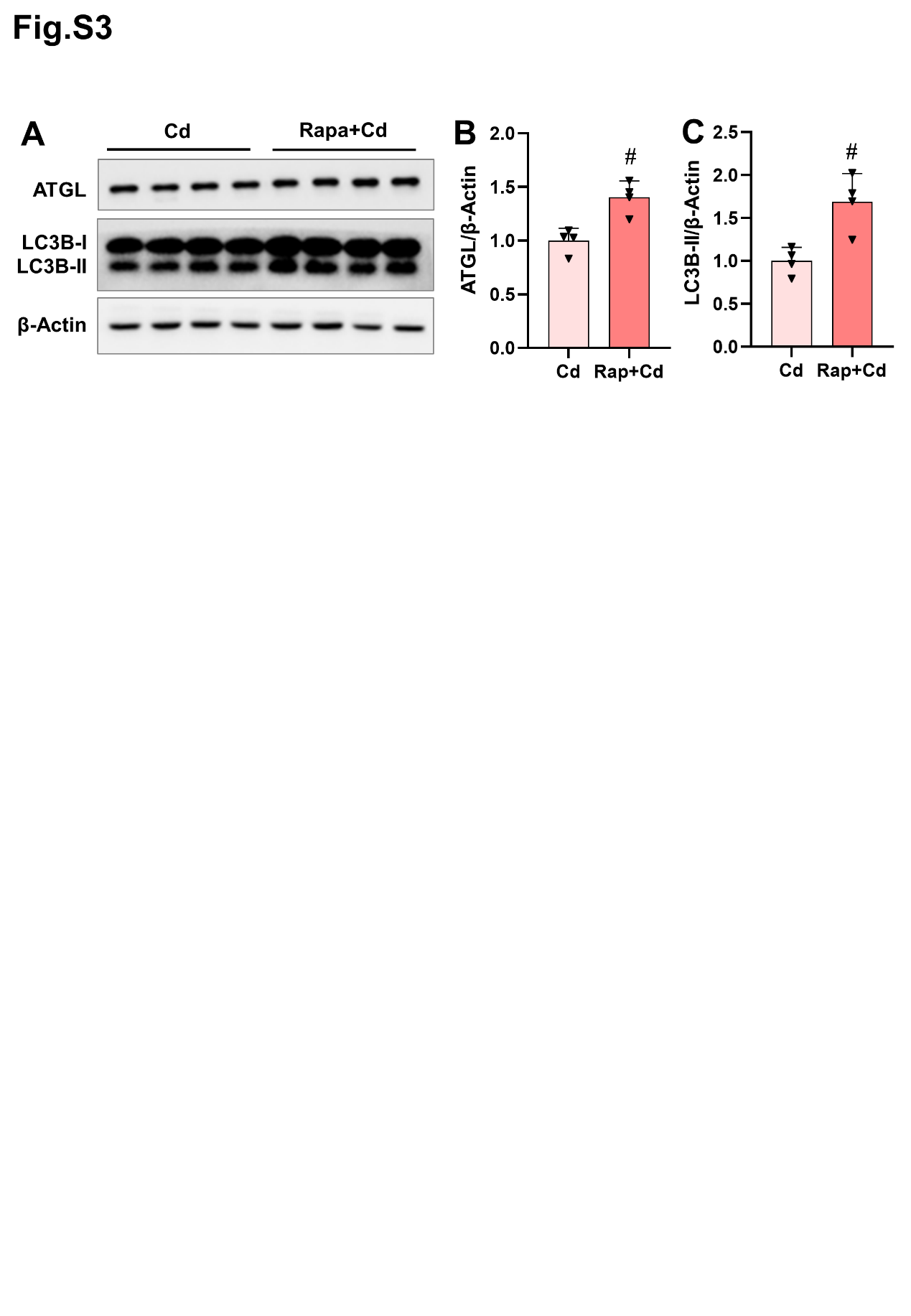


**Fig. S3. Activation of lipophagy alleviates cadmium-evoked neural tube defects in mice.** Mice were pretreated with Rap before Cd (2.0 mg/kg, i.p.) administration, placentae of Cd and Rap + Cd groups were from fetuses with NTDs. (A) Representative immunoblots of ATGL and LC3B-II proteins (n=4). (B and C) Quantification for ATGL and LC3B-II. Data were shown as *mean ± SD*. ^*^ *P* < 0.05, ^**^*P* < 0.01 compared to Ctrl group, ^#^ *P* < 0.01, ^##^ *P* < 0.01 compared to Cd (Non-NTD) group.


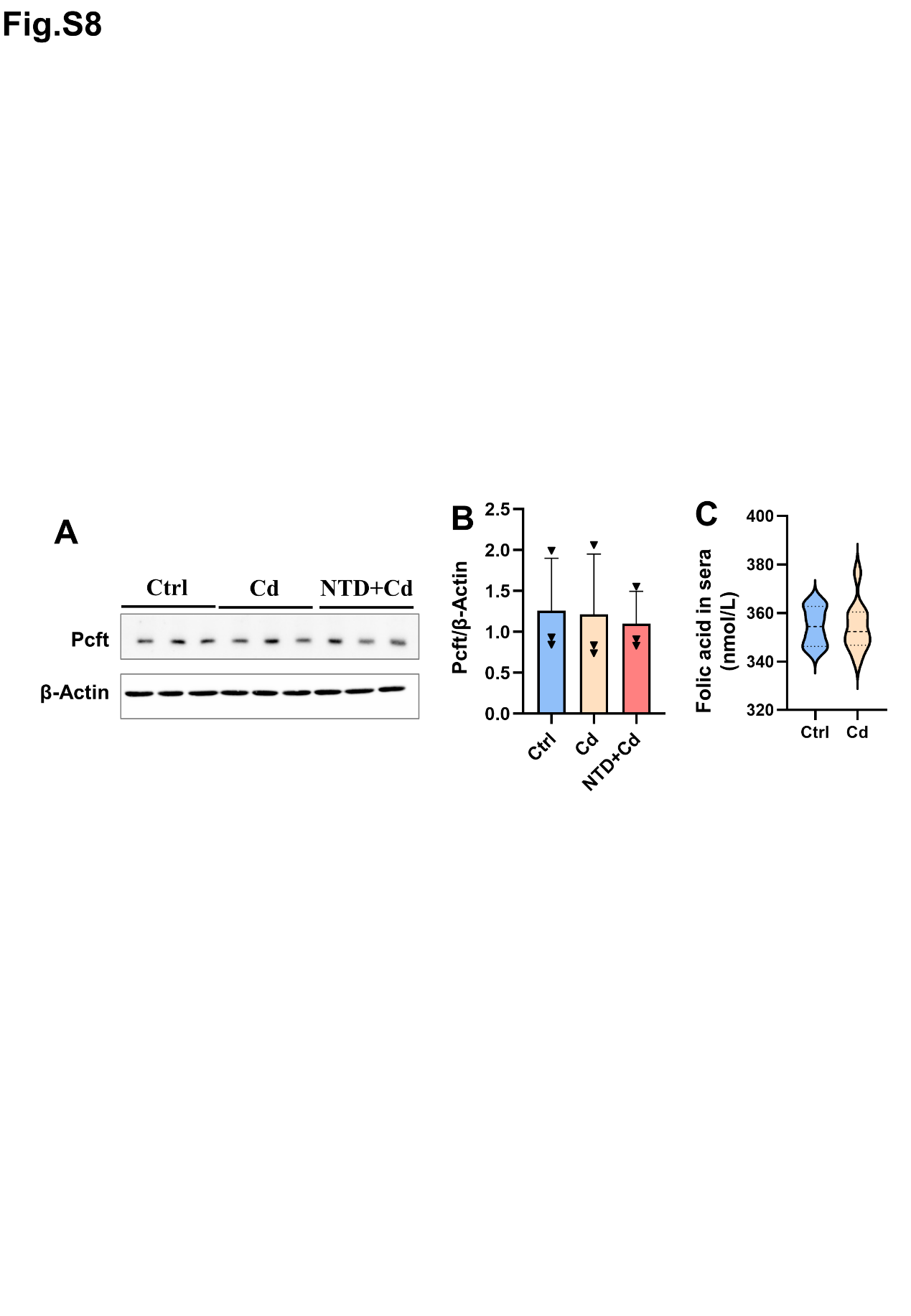


**Fig. S4.** (A) Representative immunoblots of Pcft protein (n=3). (B) Quantification for Pcft. (C) Folic acid content in the sera (nmol/L). Data were shown as *mean ± SD*. ^*^ *P* < 0.05, ^**^*P* < 0.01 compared to Ctrl group, ^#^ *P* < 0.01, ^##^ *P* < 0.01 compared to Cd (Non-NTD) group.

**Supplementary data (Plagiarism Check report)**

iThenticate: Similarity Report ID (oid:9177:59031937)
12% Overall Similarity

**
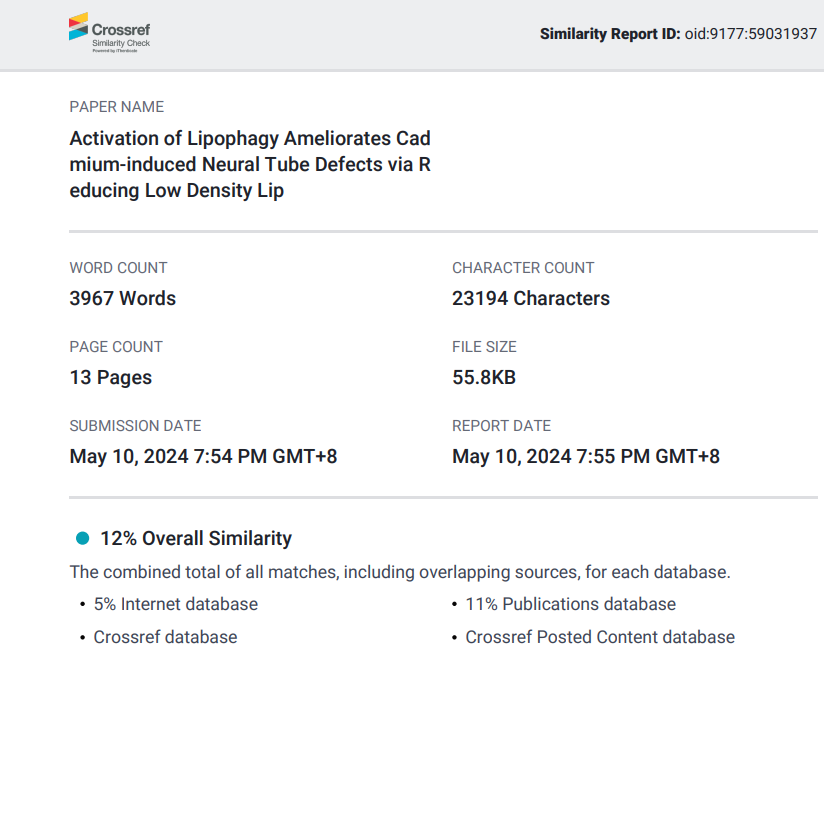
**

**References**

Dai, Y., Li, T.H., He, X., Yan, S.B., Gao, Y., and Chen, Y. (2021). The Effect and Mechanism of Asymmetric Dimethylarginine Regulating Trophoblastic Autophagy on Fetal Growth Restriction. Reprod Sci *28*, 2012-2022.

Ikeh-Tawari, E.P., Anetor, J.I., and Charles-Davies, M.A. (2013). Cadmium level in pregnancy, influence on neonatal birth weight and possible amelioration by some essential trace elements. Toxicol Int *20*, 108-112.

Liu, Y., Zhang, Y., Peng, J., Wang, H., Li, X., Li, X., Rong, X., Pan, J., and Peng, Y. (2019). Autophagy alleviates ethanol-induced memory impairment in association with anti-apoptotic and anti-inflammatory pathways. Brain Behav Immun *82*, 63-75.

Satarug, S., Garrett, S.H., Sens, M.A., and Sens, D.A. (2010). Cadmium, Environmental Exposure, and Health Outcomes. Environmental Health Perspectives *118*, 182-190.

Wang, H., Wang, Y., Bo, Q.L., Ji, Y.L., Liu, L., Hu, Y.F., Chen, Y.H., Zhang, J., Zhao, L.L., and Xu, D.X. (2016). Maternal cadmium exposure reduces placental zinc transport and induces fetal growth restriction in mice. Reprod Toxicol *63*, 174-182.

Xiong, Y.W., Zhu, H.L., Nan, Y., Cao, X.L., Shi, X.T., Yi, S.J., Feng, Y.J., Zhang, C., Gao, L., Chen, Y.H.*, et al.* (2020). Maternal cadmium exposure during late pregnancy causes fetal growth restriction via inhibiting placental progesterone synthesis. Ecotoxicol Environ Saf *187*, 109879.

Zhang, G.B., Wang, H., Hu, J., Guo, M.Y., Wang, Y., Zhou, Y., Yu, Z., Fu, L., Chen, Y.H., and Xu, D.X. (2016). Cadmium-induced neural tube defects and fetal growth restriction: Association with disturbance of placental folate transport. Toxicol Appl Pharmacol *306*, 79-85.

Zhang, Y.F., Zhu, H.L., Xu, X.F., Zhang, J., Ling, Q., Zhang, S., Chang, W., Xiong, Y.W., Xu, D.X., and Wang, H. (2023). Activation of Atg5-dependent placental lipophagy ameliorates cadmium-induced fetal growth restriction. Environ Pollut *328*, 121602.

Zhou, G.-X., Zhu, H.-L., Shi, X.-T., Nan, Y., Liu, W.-B., Dai, L.-M., Xiong, Y.-W., Yi, S.-J., Cao, X.-L., Xu, D.-X.*, et al.* (2021). Autophagy in Sertoli cell protects against environmental cadmium-induced germ cell apoptosis in mouse testes. Environmental Pollution *270*.
